# Nonclinical evaluations of deucravacitinib and Janus kinase inhibitors in homeostatic and inflammatory pathways

**DOI:** 10.3389/fimmu.2024.1437512

**Published:** 2024-09-30

**Authors:** Brandon Johnson, Lihong Cheng, Jennifer Koenitzer, Ian M. Catlett, Peter Schafer

**Affiliations:** ^1^ Translational Medicine, Immunology, Cardiovascular & Neuroscience Disease Team, Bristol Myers Squibb, Princeton, NJ, United States; ^2^ Immunology Thematic Research Centers (TRC), Bristol Myers Squibb, Princeton, NJ, United States

**Keywords:** deucravacitinib, TYK2, JAK, JAK inhibitors, tofacitinib, baricitinib, upadacitinib

## Abstract

Translational medicine provides insight into novel drugs and predicts unwanted effects. In well-characterized pathways (e.g., cytokine-Janus kinase [JAK]-signal transducers and activators of transcription [STAT]), a variety of *in vitro* assessments were used to estimate selectivity of effects on different potential targets (i.e., JAK1, JAK2, JAK3, and tyrosine kinase 2 [TYK2]). Several approved drugs were characterized as selective for the JAK family. These assessments are challenged by a lack of compounds that only inhibit one JAK family member. Deucravacitinib is a first-in-class, oral, selective, allosteric inhibitor of TYK2, a kinase required for IL-12, IL-23, and Type I interferon signaling. Unlike deucravacitinib, which selectively binds to the TYK2 regulatory domain, JAK1,2,3 inhibitors target the catalytic domain, contributing to nonselective targeting of JAK1,2,3. Cytokines associated with JAK1,2,3 signaling are required for both immune and nonimmune functions. A similar laboratory abnormality profile was observed in clinical trials using JAK1,2,3 inhibitors that has not been observed with deucravacitinib. *In vitro* testing of JAK1,2,3 inhibitors has relied upon assays of signal transduction, such as those measuring STAT phosphorylation, for estimates of potency and selectivity. These assay systems can be effective in estimating *in vivo* efficacy; however, they may not provide insight into downstream outcomes of receptor signaling, which may be more relevant for evaluating safety aspects. Assay systems assessing functional outcomes from cells may yield a more useful translational evaluation. Here, deucravacitinib was assessed for potency and selectivity versus three representatives of the JAK inhibitor class (tofacitinib, baricitinib, and upadacitinib) based on functional assays. JAK inhibitors had suppressive activity against JAK2-dependent hematopoietic colony-forming assays modeling thrombopoiesis, erythropoiesis, and myelopoiesis; however, deucravacitinib did not. Deucravacitinib had limited potency against NK cells, cytotoxic T cells, T-helper cells, and regulatory T cells activated by JAK1/JAK3-dependent common gamma chain cytokines. These data are consistent with the biologic role of JAK1,2,3 and pharmacodynamic changes in clinical laboratory abnormalities. Against TYK2-dependent cytokines, deucravacitinib selectively inhibited Type I interferon stimulation of monocytes and dendritic cells and was a more potent inhibitor than JAK inhibitors. IL-12 and IL-23 functional outputs were similarly potently inhibited by deucravacitinib. Results are consistent with deucravacitinib selectively inhibiting TYK2.

## 1 Introduction

Tyrosine kinase 2 (TYK2) is an intracellular enzyme required for signal transduction by Type I interferons (IFNs), interleukin (IL)-12/IL-23, IL-10, and Type III IFN cytokine families. TYK2 is a member of the Janus kinase (JAK) family, which includes JAK1, JAK2, and JAK3. JAKs work in obligate pairs to affect the biologic functions of the various cytokines and growth factors that use each pair. Specific biologic functions absolutely require specific cytokines and both members of a JAK pair. Kinase pairs are dictated by the cytokine receptor and the constitutively associated kinase; pairs include JAK1/JAK2, JAK1/JAK3, JAK2/JAK2, JAK1/TYK2, and JAK2/TYK2. JAK2 is used by hematopoietic cytokines and a wide range of immune and non-immune pathways (i.e., erythropoietin (EPO), IFNγ, and IL-6, respectively). Alternatively, JAK1 and JAK3 mediate signal transduction by the common gamma chain cytokines: IL-2, IL-4, IL-7, IL-9, IL-15, and IL-21. These are mediators of immune cell development, homeostasis, or activation. T-cell, natural killer (NK) cell, and regulatory T-cell development and homeostasis are controlled by IL-7 (1), IL-15 (2), and IL-2 (3), respectively. Many cytokines and growth factors utilize the JAK1/JAK2 pairing and are involved in a variety of physiologic functions (e.g., IL-6 uses this pair and is involved in inflammatory processes and lipid homeostasis).

Several drugs that target members of the JAK family are approved (4, 5). The established pharmaceutical class, JAK inhibitor, is ATP-competitive, active site, catalytic (kinase) domain binding inhibitor. Molecules of this class inhibit multiple members of the JAK family, primarily JAK1 or JAK3; however, varying degrees of JAK2 inhibition have also been observed in clinical trials. This class has a common set of laboratory changes resulting from both on-target and off-target effects. Class effects include on-target effects (i.e., immunosuppression) and off-target effects (i.e., decreased hemoglobin, decreased neutrophils, platelet modulation, liver enzyme increases, creatine phosphokinase increases, decreased lymphocytes, and cholesterol increases) (6–9). This profile can be linked to the range of JAK family members inhibited and the degree of inhibition for each family member. JAK2 is critically involved in hematopoiesis, and effects of inhibition can be observed through measures of red blood cell levels. Dose-dependent decreases in hemoglobin observed with the JAK inhibitors tofacitinib (10, 11), baricitinib (12), and upadacitinib (13) are evidence of the impact on JAK2 kinase. Thus, pharmacodynamic changes in laboratory tests reveal the *in vivo* impact of inhibition of specific JAK family members. This nonspecificity for JAK family members has limited the dose levels tolerated for optimal therapeutic effect (11). Selective inhibitors of only one family member may convey a better ratio of desired pathway targeting while sparing unwanted effects.

TYK2-dependent cytokines are critical mediators of inflammatory diseases, including IL-23 in psoriasis (14) and inflammatory bowel disease (15), or Type I IFNs in systemic lupus erythematosus (SLE) (16) and Sjögren’s syndrome (17). Partial loss of function mutations in TYK2 have been associated with protection in these disease states and others (18–20). Furthermore, complete loss of function in TYK2 results in recurrent infections linked to these cytokines but not to developmental or homeostatic dysfunction (21). Thus, TYK2 is an attractive target for therapeutic intervention. Deucravacitinib, an oral, selective, allosteric TYK2 inhibitor, is approved for the treatment of adults with moderate to severe plaque psoriasis who are candidates for systemic therapy (22, 23). Deucravacitinib uniquely binds to the regulatory (pseudokinase) domain of TYK2 rather than to the catalytic (kinase) domain where JAK1,2,3 inhibitors bind (24, 25), driving its selectivity and representing the first in a new class of oral drugs. Deucravacitinib shows no inhibition of JAK1, JAK2, or JAK3 at clinically relevant concentrations (25). In clinical trials, deucravacitinib does not have the same pharmacodynamic profile of laboratory changes and abnormalities as JAK1,2,3 inhibitors (26, 27).

JAK1,2,3 inhibitor activity is typically characterized using *in vitro* assays to measure proximal phosphorylation of signal transduction and activation of transcription (pSTAT) clinically (28–30). However, the translational value of assays used to approximate pharmacokinetic profiles and clinical effects, such as dose-limiting off-target effects of hemoglobin decrease, is unclear. Cellular assays of downstream functional outcomes, as opposed to proximal measures of kinase activity, may have greater translational value to predict a clinical profile.

In this report, deucravacitinib and approved JAK inhibitors were compared in cytokine-mediated assays representative of cellular functions. The aim of this study was to assess whether assays integrating inputs of cellular signaling with functional response might provide better estimates of the kinase inhibition profile. Assays with translational value should provide better estimates of targeted and nontargeted pathway inhibition in clinical trials and more accurately identify relevant risks and the risk-associated exposures.

## 2 Materials and methods

### 2.1 Drug compounds and dosing

Deucravacitinib and tofacitinib were synthesized internally by Bristol Myers Squibb. Baricitinib and upadacitinib were acquired from MedChemExpress (Monmouth Junction, NJ). Each compound was resuspended in 100% dimethyl sulfoxide (DMSO) (MilliporeSigma, Burlington, MA) for a 10 mM stock solution. Compounds were then prepared in 2x solutions and mixed 1:1 with media containing cells. For whole blood (WB) assays, 20x solutions in cell media were created before addition of a small volume to blood. Subsequently, a small volume (2 µL) of cytokines was added to the total volume after compound pre-incubation. Dose concentration ranged from 0.5 or 1 nM minimal concentration to 5 or 10 µM maximal concentration in an 8- to 10-point semi-log dilution.

### 2.2 PBMC functional assays

Peripheral blood mononuclear cells (PBMCs) of normal healthy volunteers (NHVs) from commercially procured leukopaks (Biological Specialty Company, Colmar, PA, and StemExpress, Folsom, CA) were processed, isolated using standard Ficoll gradient-based separation methods, and cryopreserved. On the day of the assay, cells were thawed, suspended in complete media (RPMI 1640 + 10% heat-inactivated fetal bovine serum (FBS) + 5 µg/mL gentamicin), and seeded in technical duplicate at 1-2 × 10^5^ cells per well, then rested for 1 hour. Cells were pretreated with deucravacitinib, tofacitinib, baricitinib, or upadacitinib for ~30 minutes before the addition of recombinant IL-2 (BioLegend, San Diego, CA), recombinant IL-7 (BioLegend), recombinant IL-15 (BioLegend), recombinant IFN-α2 (R&D Systems, Minneapolis, MN), recombinant IL-18 (BioLegend), recombinant IL-12 (R&D Systems), or recombinant IFNγ (R&D Systems) for 18 hours to 6 days. For measurement of proliferation, cells were labeled with CellTrace Far Red (ThermoFisher, Philadelphia, PA) and cultured for 6 days in the presence or absence of cytokines. At endpoint, cells were collected and stained for lineage and activation markers for fluorescence-activated cell-sorting (FACS) analysis. Proliferation was used as an assessment of cellular function in response to IL-15 and IL-7. BCL-2 expression was used as a surrogate marker of functional survival for IL-2− and IL-7−treated cells. The upregulation of GARP was used as representation of T-regulatory cell (Treg) functional response to IL-2. Expression of Treg lineage markers was used to confirm the maintenance/expansion of these cells in culture and was considered a functional output. Cell supernatant was collected for enzyme-linked immunosorbent assays (ELISAs). Supernatants were quantified for concentrations of CXCL9, CXCL10, CXCL11, and/or IFNγ per manufacturer’s protocol as a representation of cellular function to inflammatory JAK1/TYK2 cytokines. All ELISA kits were sourced from R&D Systems.

### 2.3 Phosphorylation of STAT signaling assays

PBMCs were isolated via a Ficoll gradient from human WB drawn into ethylenediaminetetraacetic acid (EDTA) tubes. PBMCs, which were obtained from NHV at Bristol Myers Squibb with approval of Bristol Myers Squibb Environmental Health & Safety and with written informed consent from the donors, were used for pSTAT assays. Additional pSTAT assays were performed on PBMCs from NHV of commercial leukopaks. Here, cryopreserved PBMCs were thawed in RPMI 1640 containing 10% FBS at 37°C before assay stimulation. After a 1-hour rest period, PBMCs or WB samples were preincubated with inhibitors at least 20 minutes prior to the addition of recombinant IFNα (PBL Assay Science, Piscataway, NJ), recombinant thrombopoietin (TPO; PeproTech, Cranbury, NJ), recombinant IL-7, or recombinant IL-15. After 15 minutes, cell stimulation was stopped with warm Lyse/Fix Buffer before proceeding to FACS staining.

### 2.4 Monocyte-derived dendritic cell functional assays

Leukopaks from NHV were commercially purchased and negatively selected for CD14+ monocytes (StemCell Technologies, Vancouver, BC) and cryopreserved. Monocytes were thawed in RPMI 1640 + 10% FBS at 1 × 10^6^ cells/mL and rested for 1 hour at 37°C. Six million monocytes were plated in six-well dishes with 100 ng/mL IL-4 (Miltenyi Biotec, Gaithersburg, MD) and 100 ng/mL granulocyte-macrophage colony-stimulating factor (GM-CSF; Miltenyi Biotec) and incubated for 6 to 7 days. Cells were fed fresh media with additional IL-4 and GM-CSF every 3 days prior to assay. Non-adherent immature monocyte-derived dendritic cells were collected, washed, and resuspended at 1 × 10^6^ cells/mL in complete media. The 1 × 10^5^ cells were plated in technical duplicate wells and a nine-point, semi-log dilution of deucravacitinib, tofacitinib, baricitinib, or upadacitinib was added ~30 minutes prior to incubation with recombinant IFN-α2 for 48 hours. At Day 2, cell supernatants were removed for quantification of CXCL9 and CXCL10 by ELISA.

### 2.5 NK-cell functional assays

Cryopreserved PBMCs were thawed and suspended in RPMI 1640 + 10% FBS and rested for 1 hour at 37°C. NK cells were isolated by negative bead selection (StemCell Technologies) in accordance with the manufacturer’s protocol. Purified NK cells were counted, centrifuged, and seeded in technical duplicate at 1 × 10^5^ cells per well. A nine-point, semi-log dilution of deucravacitinib, tofacitinib, baricitinib, or upadacitinib was added to the cells for preincubation before the addition of recombinant IL-23 (BioLegend) and recombinant human IL-1β (R&D Systems). Cells were collected at 18 hours and stained for lineage and activation marker for FACS analysis. Supernatants were collected for quantification of IFNγ by ELISA.

### 2.6 Functional assay staining and FACS analysis

For functional assays, cells were washed twice with Dulbecco’s phosphate buffered saline (DPBS) and resuspended in 50 µL PBS containing 1:1000 diluted Fixable Near-IR Dead Cell Stain Kit (ThermoFisher) for 30 minutes. Cells were washed twice in Cell Staining Buffer (BioLegend) and stained with FITC CD3 (clone OKT3; eBiosciences, San Diego, CA), Brilliant™ Blue (BB)700 CD3 (clone HIT3α; BD Biosciences, Franklin Lakes, NJ), Brilliant™ Violet (BV)510 CD56 (clone HP-3G10; BD Biosciences), BV786 NKG2D (clone 1D11; BioLegend), PE NKp30 (clone p30-15; BD Biosciences), TruStain FcX (BioLegend), BV421 CD107a (clone R35-38; BD Biosciences), Brilliant™ UltraViolet (BUV)395 CD8 (clone RPA-T8; BD Biosciences), BV510 CD56 (clone 5.1H11; BioLegend), BUV496 CD4 (clone SK3; BD Biosciences), BB515 CD45RO (clone UCHL1; BD Biosciences), BV786 CD25 (clone M-A251; BD Biosciences), BV421 CCR7 (clone G043H7; BioLegend), APC CD127 (clone 40131; R&D Systems), PE-Cy7 CD3 (clone UCHT1; BD Biosciences), BB700 CD45RO (clone UCHL1; BD Biosciences), BV421 CD4 (clone RPA-T4; BD Biosciences), PE CD8 (clone RPA-T8; BD Biosciences), V450 CD14 (clone M5E2; BD Biosciences), APC CD11c (clone BU15; eBioscience), PE CD80 (clone 2D10.4; eBioscience), V500 HLA-DR (clone G46-6; BD Biosciences), BUV395 CD40 (clone 5C3; BD Biosciences), BB515 CD54 (clone HA58; BD Biosciences), BV785 CD19 (clone HIB19; BioLegend), CD69 (clone FN50; BD Biosciences), PE-CF594 CD56 (clone NCAM16.2; BD Biosciences), BUV395 GARP (clone 7B11; BD Biosciences), BV421 NKG2D (clone 1D11; BioLegend), BV480 NKp30 (clone p30-15; BD Biosciences), BUV395 CD69 (clone FN50; BD Biosciences), BV510 CD19 (clone HIB19; BioLegend), BV605 CD8 (clone RPA-T8; BioLegend), BV605 NKp30 (clone p30-15; BD Biosciences), PE CD218a (clone H44; BioLegend), APC CD56 (clone NCAM16.2; BD Biosciences), FITC CD69 (clone FN50; BD Biosciences), BV421 CD14 (clone M5E2; BD Biosciences), BV711 CD11c (clone B-ly6; BD Biosciences), or BV605 CD86 (clone 2331 [FUN-1]; BD Biosciences) for 30 minutes in Cell Staining Buffer containing Brilliant Stain Buffer (BD Biosciences). In instances of intracellular staining, cells were washed and resuspended in FOXP3 Transcription Factor Fixation Buffer (ThermoFisher) for 30 minutes. Cells were washed twice with Permeabilization Buffer (ThermoFisher) before resuspension in Permeabilization Buffer containing PE BCL-2 (clone Bcl-2/100; BD Biosciences), AF647 FOXP3 (clone 236A/E7; BD Biosciences), PE-CF594 FOXP3 (clone 236A/E7; BD Biosciences), AF647 T-bet (clone 04-46; BD Biosciences), and/or PE-CF594 T-bet (clone 04-46; BD Biosciences) for 30 minutes. Cells were washed twice and resuspended in Cytofix Fixation Buffer (BD Biosciences) for 30 minutes. Cells were washed and resuspended in Cell Staining Buffer before flow cytometry analysis on an LSRFortessa.

### 2.7 Signaling assay staining and FACS analysis

For signaling assays, after treatment with inhibitor and/or cytokine, stimulation was stopped by thoroughly mixing cells or WB with 3x volume of Lyse/Fix Buffer (BD Biosciences) for 10 minutes at 37°C. Fixed cells were then washed with Cell Staining Buffer twice and stained with surface antibodies of TruStain FcX, FITC CD61 (clone VI-PL2; BD Biosciences), PE-Cy7 CD3, BB700 CD45RO, BV786 CD25, BV421 CD4, and/or PE CD8 for 30 minutes in Cell Staining buffer at room temperature. Cells were washed twice with Cell Staining Buffer before the addition of 3x volume of ice-cold Permbuffer III (BD Biosciences). Cells were incubated on ice for 30 minutes, then washed twice with 3x volume of Cell Staining Buffer. PE pSTAT3 (clone 4/P-STAT3; BD Biosciences), AF647 pSTAT5 (clone 47/Stat5(pY694); BD Biosciences), AF647 pSTAT2 (clone D3P2P; Cell Signaling), AF488 FOXP3 (clone 259D/C7; BD Biosciences), and/or PE-CF594 CD56 (clone NCAM16.2; BD Biosciences) in Cell Staining Buffer were incubated with cells for 30 minutes at room temperature. Cells were washed twice and resuspended in Cytofix Fixation Buffer before flow cytometry analysis on an LSRFortessa. The amount of pSTAT expression was quantified by median fluorescence intensity (MFI) after gating on parent population and comparing with fluorescent minus one and unstimulated samples. Gating strategy of cell populations and example plots of functional (**Supplementary Figures 1A, B, D, E**) and signaling (**Supplementary Figure 1C**) expression are provided.

### 2.8 Hematopoietic differentiation assays

Cryopreserved normal human bone marrow light density cells (Lonza Bioscience, Walkersville, MD, and StemCell Technologies) were thawed into Iscove’s Modified Dulbecco’s Medium + 2% FBS containing DNase I (StemCell Technologies). Cells were washed and resuspended in Iscove’s Modified Dulbecco’s Medium + 2% FBS and counted. For erythroid and myeloid progenitor evaluation, 1-1.5 × 10^4^ cells were suspended in MethoCult™ media (StemCell Technologies) containing eight-point dilutions of deucravacitinib, tofacitinib, baricitinib, or upadacitinib. Cell mixtures were then plated into SmartDish™ for 13 to 15 days at 37°C. Following this incubation period, erythroid and myeloid progenitors were evaluated for morphology by the STEMvision™ automated colony-forming unit assay reader and scored. For megakaryocyte progenitor evaluation, 1-1.5 × 10^5^ cells were suspended in MegaCult-C™ media (StemCell Technologies) containing eight-point dilutions of deucravacitinib, tofacitinib, baricitinib, or upadacitinib. Collagen was added to cell mixtures, then plated onto double chamber slides and incubated at 37°C. After 10 to 12 days, slides were dehydrated, fixed, and stained with anti-CD41 (StemCell Technologies). Cells were then enumerated and scored based on size and morphology.

### 2.9 IC_50_ calculation and modeling WB potency

Percent inhibition was determined by the following equation:


$$\%\;\text{Inhibition}=\left(1-\left(\frac{\text{Kcon}-\text{Un}}{\text{Ct}-\text{Un}}\right)\right)*100$$

K_con_ is the readout of cytokine-stimulated cells with kinase inhibitor at any given concentration, U_n_ is the readout for the DMSO vehicle control, and C_t_ is the readout for the cytokine stimulation with DMSO vehicle. Half-maximal inhibitory concentration (IC_50_) values were determined by curve fitting using nonlinear regression analysis on GraphPad Prism for each donor’s response. Zero inhibition was set at 10^-11^ M. Model was constrained to maximal inhibition (100% of treatment control) only when appropriate. IC_50_ was determined by the geometric mean of the donors. The WB IC_50_ values for the inhibitors from measured *in vitro* culture media IC_50_ was determined using the following equation (30):


$$\text{WB}\;\text{I}{\text{C}}_{50}=\frac{\left(\left(\text{Blood}:\text{Plasma}\;\text{Ratio}\right)*\left(\text{CM}\;\text{I}{\text{C}}_{50}\right)\right)}{{\text{f}}_{\text{u}}}$$

The unbound free fraction (f_u_) for deucravacitinib was 0.184 and the blood:plasma ratio was 1.26. The f_u_ for tofacitinib, baricitinib, and upadacitinib were 0.61, 0.59, and 0.56, respectively (30). The blood:plasma ratio for tofacitinib, baricitinib, and upadacitinib were 1.20, 1.32, and 1.16, respectively. Possible serum-bound fraction in *in vitro* culture medium containing FBS was not used in the determination of culture media IC_50_.

### 2.10 Statistical analysis

A statistical analysis was performed using GraphPad PRISM 9. One-way ANOVA was performed to determine differences between groups. Each JAK1,2,3 inhibitor matched donor-to-donor response was compared to deucravacitinib using a paired two-tailed Student’s t test. Data are represented by geometric mean of at least two independent experiments containing multiple donors, where the ‘n’ is representative of donor numbers. Error bars represent ± standard error of the mean and *P* values are indicated by *P < 0.05, **P < 0.01, ***P < 0.001.

## 3 Results

Clinically relevant dose must be considered when framing the effect of a kinase inhibitor on a given pathway. Here, we classified inhibitors as being highly potent if the IC_50_ was determined to be less than or equal to the maximal WB drug concentration (C_max_) of the highest approved maintenance dose (28). Modest potency was considered IC_50_ <3x C_max_, mild potency was considered <5x C_max_, and minimal to no potency was considered >5x C_max_.

**Figure 1:**
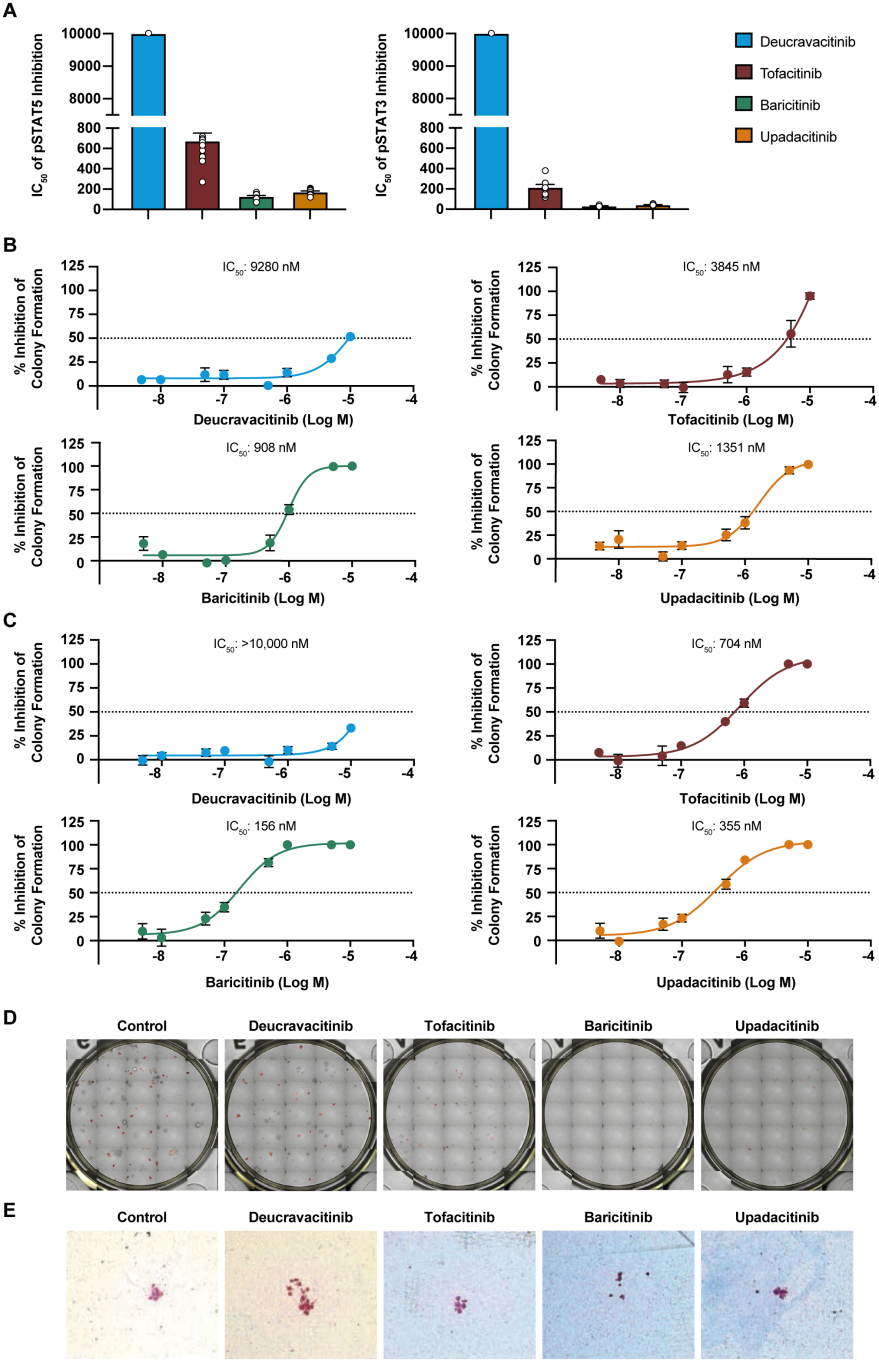
JAK inhibitors, but not deucravacitinib, have potency in JAK2-reliant hematopoietic assays. **(A)** Measured IC_50_ values of kinase inhibitors against thrombopoietin (50 ng/mL)-induced pSTAT5 and pSTAT3 of platelets from normal healthy volunteer human whole blood (n = 6-12 donors). Note that pSTAT3 data are adapted from Chimalakonda et al. (28), and graphed here for comparison. **(B)** Dose titration of kinase inhibitors on total megakaryocyte progenitor colony formation (Total CFU-Mk) from human pluripotent stem cell-cultured MegaCult™ containing thrombopoietin (50 ng/mL), IL-3 (10 ng/mL), and IL-6 (10 ng/mL) for 10 to 12 days (n = 4 donors). **(C)** Dose titration of kinase inhibitors on total erythroid progenitor colony formation from human pluripotent stem cell-cultured in MethoCult™ containing stem cell factor, GM-CSF, IL-3, G-CSF, and thrombopoietin for 13 to 15 days. **(D)** Representative images of total colony forming cluster (Total CFC) in MethoCult™ colony formation at 500 nM of kinase inhibitors. **(E)** Representative images of small CFU in MegaCult™ cultures at 1000 nM of kinase inhibitors.

Deucravacitinib does not bind to or inhibit JAK2 in biochemical assays (25) and STAT phosphorylation assays (24). Similarly in the present study, there was no effect of deucravacitinib against pSTAT3 or pSTAT5 signaling in TPO-stimulated platelets (**Figure 1A**). The JAK1/JAK3 inhibitor tofacitinib had modest to high potency against pSTAT5 (IC_50_ = 628 nM) and pSTAT3 (IC_50_ = 201 nM). Baricitinib, known to be potent against JAK1 and JAK2 (31), was highly potent against pSTAT5 (IC_50_ = 124 nM) and pSTAT3 (IC_50_ = 31 nM) signaling in this assay. Upadacitinib, a reported JAK1 inhibitor, was also highly potent (pSTAT5 IC_50_ = 169 nM; pSTAT3 IC_50_ = 40 nM) in this signaling assay (**Figure 1A**). Functional models of megakaryocyte differentiation may improve the predictive power of *in vitro* inhibition assays. Hematopoietic stem cells differentiate into megakaryocyte progenitors with the addition of a mixture of JAK2-dependent cytokines that includes TPO. Unlike TPO pSTAT signaling, differentiation assays were less sensitive to the JAK inhibitors tofacitinib, baricitinib, and upadacitinib by only minimally inhibiting cell differentiation. Deucravacitinib consistently did not inhibit either signaling or functional assays of TPO (**Figure 1B**; **Table 1**). Hematopoietic stem cells stimulated with a cocktail of JAK2 cytokines induced differentiation into erythroid, myeloid, or granulocyte progenitors. It was previously reported that JAK1,2,3 inhibitors, but not deucravacitinib, were highly potent against EPO signaling in an erythroleukemic cell line (24). Here, erythroid progenitors were not observed to be functionally inhibited by deucravacitinib at concentrations exceeding 10 µM. Tofacitinib (IC_50_ = 704 nM), baricitinib (IC_50_ = 156 nM), and upadacitinib (IC_50_ = 355 nM) had modest potency (**Figure 1C**; **Table 1**). No loss of erythroid, myeloid, or granulocyte colony forming clusters (CFCs) at concentrations <500 nM, or loss in megakaryocyte colony forming units (CFUs) at concentrations <1000 nM were seen in deucravacitinib cultures. In contrast, fewer CFCs and CFUs in tofacitinib, and sparse CFCs and CFUs observed in baricitinib and upadacitinib at these concentrations (**Figures 1D, E**).

**Table 1:** Potencies of inhibitors in JAK2 hematopoietic assays.

Kinase pairing	Cytokine stimulation	Endpoint	Compound	IC_50_ (nM)	95% CI (nM)	*P* value	Calculated WB IC_50_ (nM)	n
JAK2/JAK2	IL-3, EPO, G-CSF, GM-CSF, stem cell factor	Total erythroid	Deucravacitinib	>10000	NA	NA	>10000	4
			Tofacitinib	704	523-947	0.0001	1385	4
			Baricitinib	156	140-175	0.0001	349	4
			Upadacitinib	355	213-592	0.0002	735	4
		Total CFC	Deucravacitinib	7945	5345->10000	NA	>10000	4
			Tofacitinib	637	420-968	0.0001	1253	4
			Baricitinib	162	132-200	0.0001	362	4
			Upadacitinib	237	164-341	0.0001	491	4
JAK2/JAK2 JAK1/JAK2	TPO, IL-3, IL-6	Small CFU-Mk	Deucravacitinib	>10000	NA	NA	>10000	4
			Tofacitinib	6706	3359- >10000	0.1631	>10000	4
			Baricitinib	1829	639-5234	0.0143	4092	4
			Upadacitinib	3013	1537-5905	0.0108	6241	4
		Total CFU-Mk	Deucravacitinib	9280	8492->10000	NA	>10000	4
			Tofacitinib	3845	1658-8914	0.0464	7564	4
			Baricitinib	908	635-1299	0.0002	2032	4
			Upadacitinib	1351	670-2725	0.0029	2799	4

BFU-E, burst forming unit erythroid; CFC, colony forming cluster; CFU-Mk, colony forming unit megakaryocyte; CI, confidence interval; EPO, erythropoietin; G-CSF, granulocyte colony stimulating factor; GM-CSF, granulocyte-macrophage colony-stimulating factor; IL, interleukin; JAK, Janus kinase; NA, not applicable; TPO, thrombopoietin; WB, whole blood.

**Figure 2:**
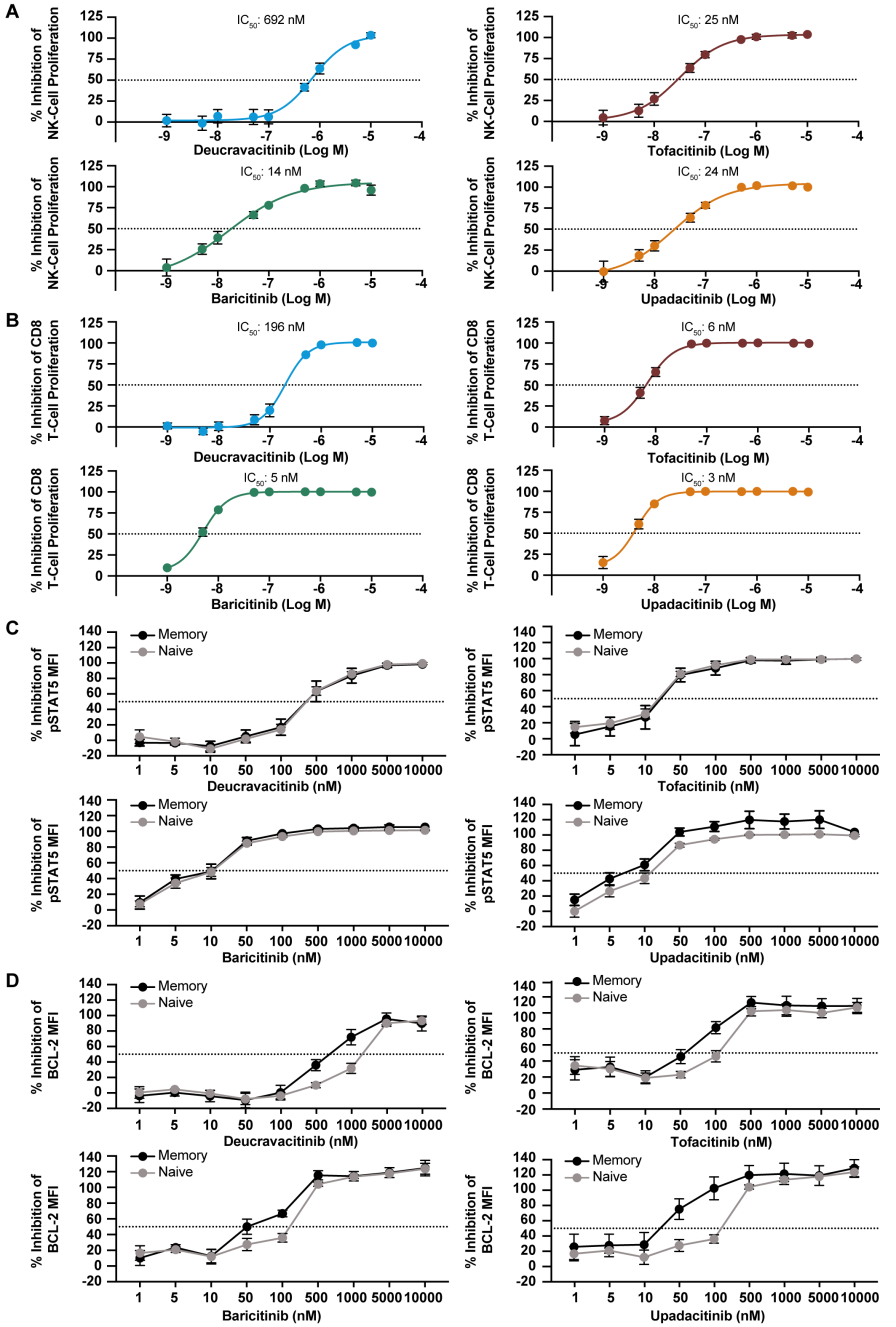
Homeostatic gamma chain cytokines are inhibited by JAK inhibitors but not deucravacitinib. PBMCs from normal healthy volunteers were treated with IL-15 (10/ng/mL) for 6 days in the presence or absence of dose-ranging kinase inhibitor. Inhibitory effect on proliferation was evaluated in: **(A)** NK cells and **(B)** CD8^+^ T cells (n = 6 donors). PBMCs from normal healthy volunteers were treated with IL-7 (50 ng/mL) for 15 min **(C)** or overnight **(D)** in the presence or absence of dose-ranging kinase inhibitor. Changes in BCL-2 and pSTAT5 median fluorescent intensity (MFI) were used to evaluate functional inhibitory effect in memory and naive CD4^+^ T cells (n = 5-6 donors).

JAK1 and JAK3 are required for the function of common gamma chain cytokines, IL-2, IL-4, IL-7, IL-9, IL-15, and IL-21. IL-7, IL-15 and IL-2 have vital homeostatic functions in T cells and NK cells, and IL-15 plays a role in NK-cell development (32) and CD8 function (33–35). IL-15–induced proliferation of NK and CD8 T cells that was strongly inhibited by JAK1,2,3 inhibitors and was only mildly to modestly inhibited by deucravacitinib (**Figures 2A, B**). IL-7 is required for thymic T-cell development (36), and T-cell survival in the periphery through expression of anti-apoptotic factor BCL-2 (37). Thus, BCL-2 expression was used as a marker of functional response to IL-7. Deucravacitinib had modest to no potency against IL-7, although differences were observed between the functional and signaling readouts in total CD4 T cells (**Table 2**). JAK1,2,3 inhibitors were highly potent against IL-7 and were consistently more potent in pSTAT5 signaling assays (**Figure 2C**) than against assays measuring BCL-2 upregulation (**Figure 2D**; **Table 2**). This observation was similarly reflected in IL-15 assays of NK cell activation by NKG2D upregulation compared with pSTAT5 expression (**Table 2**). We also studied the differences in kinase inhibitor potency against different immune cells and subsets. Interestingly, we find examples where differences between immune cell subsets are observed in common functional outputs but not in signaling outputs. In **Figure 2C**, inhibition of IL-7–mediated STAT phosphorylation was consistent in different subpopulations of CD4 T cells for each compound. Alternatively, BCL-2 expression was more potently inhibited in memory T cells than in STAT phosphorylation. Functional expression assays of BCL-2 within these donors show memory cells to be more prone to kinase inhibition than naive cells (**Figure 2D**; **Table 2**).

**Table 2:** Potencies of inhibitors common to gamma chain cytokine assays.

Kinase pairing	Cytokine stimulation	Endpoint	Compound	IC_50_ (nM)	95% CI (nM)	*P* value	Calculated WB IC_50_ (nM)	n
JAK1/JAK3	IL-15	CD56+ NK-cell pSTAT5	Deucravacitinib	800	396-1619	NA	5478	6
			Tofacitinib	16	2-152	0.0059	32	6
			Baricitinib	9	1-100	0.0042	20	6
			Upadacitinib	6	0.4-76	0.0044	12	6
		CD56+ NK-cell proliferation	Deucravacitinib	692	533-899	NA	4739	6
			Tofacitinib	25	10-61	0.0001	49	6
			Baricitinib	14	2-79	0.002	31	6
			Upadacitinib	24	13-46	0.0001	50	6
		CD56+ NK-cell NKG2D expression	Deucravacitinib	4580	3376-6213	NA	>10000	5
			Tofacitinib	136	52-359	0.0002	268	5
			Baricitinib	128	43-379	0.0004	286	5
			Upadacitinib	113	60-213	0.0001	234	5
		CD8 T-cell proliferation	Deucravacitinib	196	133-289	NA	1342	6
			Tofacitinib	6	3-11	0.0001	12	6
			Baricitinib	5	4-6	0.0001	11	6
			Upadacitinib	3	2-6	0.0001	6	6
	IL-7	Total CD4 T-cell pSTAT5	Deucravacitinib	305	152-612	NA	2089	6
			Tofacitinib	18	10-34	0.0001	35	6
			Baricitinib	10	6-18	0.0001	22	6
			Upadacitinib	9	5-17	0.0001	19	6
		Total CD4 T-cell BcL-2 expression	Deucravacitinib	900	684-1184	NA	6163	6
			Tofacitinib	97	73-129	0.0001	191	5
			Baricitinib	113	73-176	0.0002	253	6
			Upadacitinib	58	33-102	0.0001	120	5
		Memory CD4 BcL-2 expression	Deucravacitinib	551	418-726	NA	3773	6
			Tofacitinib	57	29-114	0.0015	112	5
			Baricitinib	70	42-115	0.0004	157	6
			Upadacitinib	29	15-55	0.0001	60	5
		Naive CD4 BcL-2 expression	Deucravacitinib	1486	1020-2163	NA	10176	6
			Tofacitinib	138	104-184	0.0001	272	5
			Baricitinib	190	152-237	0.0001	425	6
			Upadacitinib	108	74-158	0.0004	224	5
		Naive CD4 T-cell pSTAT5	Deucravacitinib	322	207-501	NA	2205	6
			Tofacitinib	16	8-30	0.0001	32	6
			Baricitinib	11	6-18	0.0001	25	6
			Upadacitinib	10	6-19	0.0001	21	6
		Memory CD4 T-cell pSTAT5	Deucravacitinib	264	112-623	NA	1807	6
			Tofacitinib	16	7-36	0.0001	32	6
			Baricitinib	9	5-19	0.0001	20	6
			Upadacitinib	8	4-17	0.0001	17	6
	IL-2	CD56+ NK-cell BcL-2 expression	Deucravacitinib	711	548-921	NA	4869	6
			Tofacitinib	35	21-57	0.0001	69	6
			Baricitinib	18	11-29	0.0001	40	6
			Upadacitinib	9	5-18	0.0001	19	6
		Treg lineage expression	Deucravacitinib	1397	607-3219	NA	9566	6
			Tofacitinib	18	8-42	0.0001	35	6
			Baricitinib	7	4-14	0.0001	16	6
			Upadacitinib	2	1-6	0.0001	4	6
		Treg BcL-2 expression	Deucravacitinib	3391	2263-5082	NA	>10000	6
			Tofacitinib	27	12-61	0.0001	53	6
			Baricitinib	19	13-29	0.0001	43	6
			Upadacitinib	9	4-22	0.0001	19	6
		Treg GARP expression	Deucravacitinib	1054	382-2909	NA	7218	6
			Tofacitinib	11	5-24	0.0003	22	6
			Baricitinib	7	4-11	0.0001	16	5
			Upadacitinib	4	2-10	0.0001	8	6

CI, confidence interval; IL, interleukin; JAK, Janus kinase; NA, not applicable; NK, natural killer; pSTAT, phosphorylation of signal transduction and activation of transcription; Treg, regulatory T cell; WB, whole blood.

**Figure 3:**
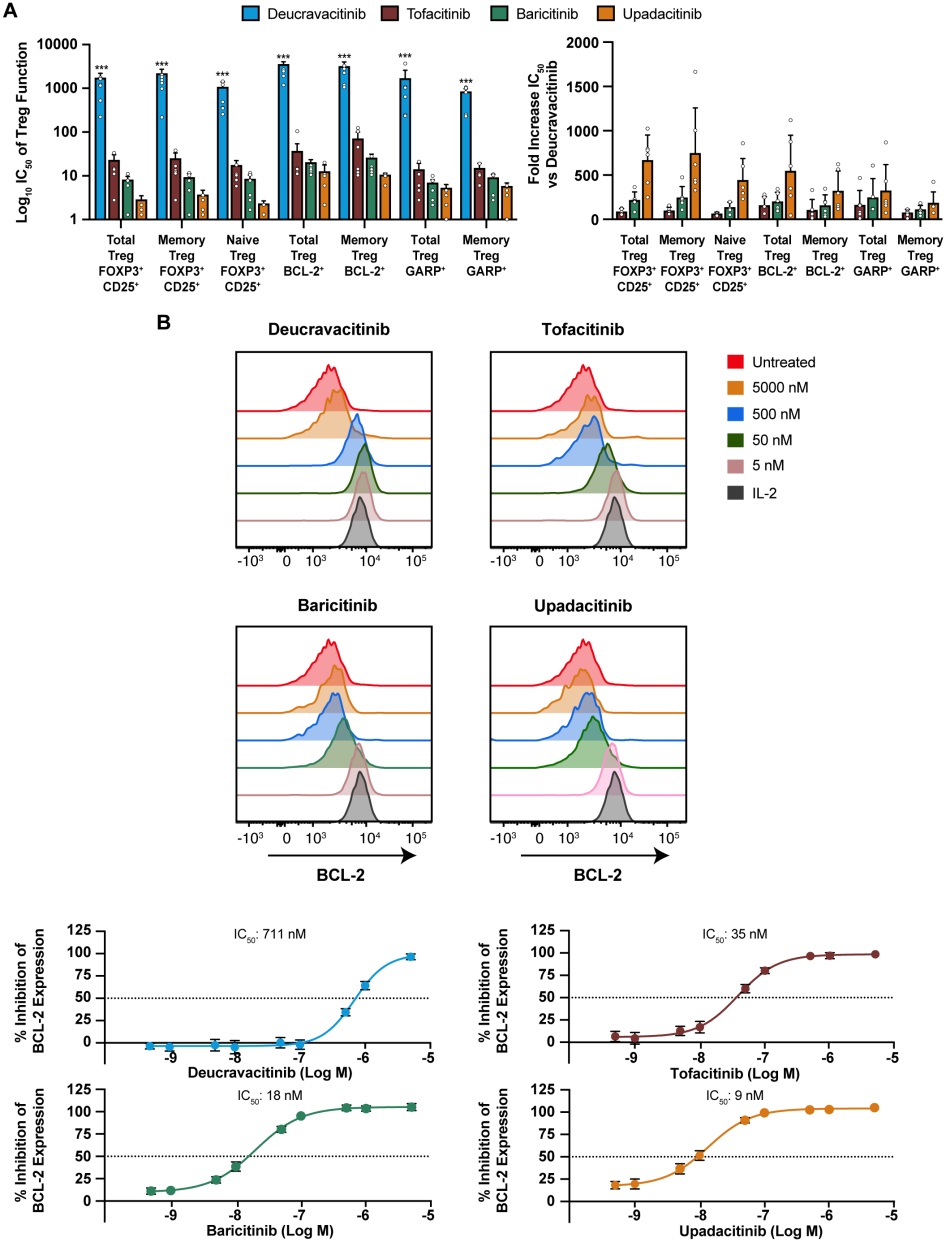
IL-2 effects in Treg and NK cells are potently inhibited by JAK inhibitors but not deucravacitinib. PBMCs from normal healthy volunteers were treated with low-dose IL-2 (2 ng/mL) for 6 days in the presence or absence of dose-ranging kinase inhibitor. **(A)** IC_50_ values were determined against functional expression and activation markers and fold difference in potency from deucravacitinib in Treg (n = 5-6 donors). Tregs were delineated as CD3^+^CD4^+^FOXP3^+^CD25^+^. Memory Tregs or naive Tregs were determined by the presence or absence of CD45RO. **(B)** Representative histograms of CD3^–^CD56^+^ NK BCL-2 response to PBMCs treated for 6 days with low-dose IL-2 in the presence of decreasing kinase inhibitor (n = 6 donors). Statistical significance of deucravacitinib from each JAK1,2,3 inhibitor was determined by paired two-tailed *t*-test. ***P < 0.001.

IL-2 is an effector cytokine for T cells (38) and an important NK-cell survival factor (39) but also necessary for Treg survival and the restraint of lethal autoimmunity (40, 41). Deucravacitinib and JAK1,2,3 inhibitors were evaluated against functional effects of IL-2 on Treg. GARP is upregulated upon Treg activation and enhances suppressive function (42). Other representative indicators of IL-2 function on Treg are BCL-2 expression and maintenance of lineage markers expression. Deucravacitinib did not inhibit survival, activation, or lineage marker expression of Tregs, whereas JAK1,2,3 inhibitors potently inhibited IL-2 function in Tregs (**Table 2**; **Supplementary Table 1**). A large magnitude of difference in potency was observed between deucravacitinib and JAK1,2,3 inhibitors, particularly upadacitinib, which was >158-fold more potent than deucravacitinib against all measured Treg outputs (**Figure 3A**). Similarly, IL-2 activity was potently inhibited in NK cells by JAK1,2,3 inhibitors (**Figure 3B**; **Table 2**).

**Figure 4:**
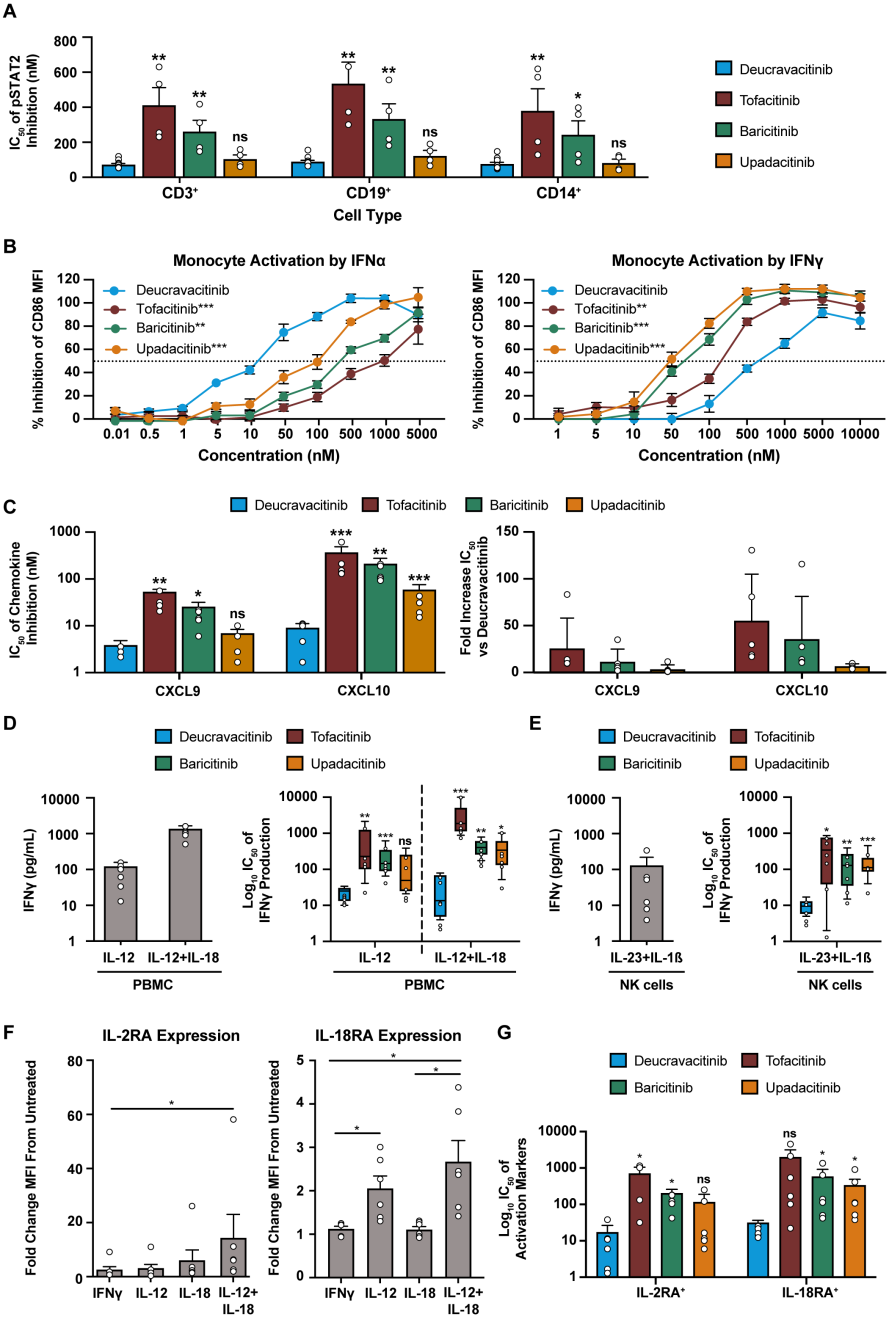
Deucravacitinib potently inhibits functional assays of Type I and Type II IFN. **(A)** Measured IC_50_ values of kinase inhibitors against IFNα (1000 U/mL)-induced pSTAT2 of CD3^+^ T cells, CD19^+^ B cells, and CD14^+^ monocytes from normal healthy volunteer human WB (n=4-12 donors). **(B)** PBMCs from normal healthy volunteers were treated with IFNα (1000 U/mL) or IFNγ (25 ng/mL) overnight in the presence or absence of dose-ranging kinase inhibitor and measured for the inhibition of CD86 expression by median fluorescence intensity on CD14^+^ monocytes (n = 6 donors). **(C)** Monocytes differentiated into monocyte-derived dendritic cells (mDC) were treated with IFNα (1000 U/mL) for 48 hours in the presence or absence of dose-ranging kinase inhibitors and measured for chemokine inhibition. IC_50_ values are represented in fold decreased potency from deucravacitinib (n = 5 donors). **(D)** PBMCs from normal healthy volunteers were treated with IL-12 (10 ng/mL) or IL-12 (2 ng/mL) + IL-18 (5 ng/mL)overnight in the presence or absence of dose-ranging kinase inhibitor and measured for the inhibition of IFNγ. **(E)** Negatively selected CD56^+^ NK cells from normal healthy volunteers were treated with IL-23 (50 ng/mL) + IL-1β (10 ng/mL) overnight in the presence or absence of dose-ranging kinase inhibitor and measured for the inhibition of IFNγ. **(F)** PBMCs from normal healthy volunteers were treated with indicated cytokines at described doses overnight. Gated CD56^+^ NK cells were evaluated for activation markers. **(G)** IL-12 + IL-18 treated PBMC cultures were additionally titrated with kinase inhibitor and evaluated for inhibitory potential against the activation markers of gated CD56^+^ NK cells. Statistical significance for expression analysis was determined by one-way ANOVA and significance of deucravacitinib from each JAK1,2,3 inhibitor was determined by paired two-tailed *t*-test. *P<0.05, **P<0.01, ***P<0.001. ns, non-significant.

*In vitro* assays are performed in culture media with limited plasma proteins and do not account for the free fraction of drug in WB. Conversions of culture media IC_50_ to WB equivalents are required for adequate estimates of *in vivo* pharmacodynamic effect. IL-7 signaling assays were used as a benchmark to test the accuracy of WB conversions of kinase inhibitors (**Supplementary Table 2**). Inhibitory potency on JAK2 and common gamma chain assays with culture media and WB conversions are summarized in **Tables 1**, **2**, respectively. Collectively, these data show that deucravacitinib has minimal to no potency against signaling or functional assays of JAK2 and common gamma chain in WB. JAK inhibitors show lesser potency in functional JAK2 assays in contrast to high potency in signaling assays and modest to high potency against common gamma chain pathways. Further evaluations of deucravacitinib and JAK1,2,3 inhibitor potencies are shown in **Supplementary Table 1**. IFNα (JAK1/TYK2) is a proinflammatory cytokine important for antiviral response and is dysregulated in several immune-mediated diseases, including SLE (43). We have previously observed some differences in potency against IFNα pSTAT assays in PBMCs favoring deucravacitinib, which targets TYK2, over tofacitinib, baricitinib, and upadacitinib, which target JAK1 (24). Testing of WB IFNα pSTAT2 supports this observation, with deucravacitinib showing up to a 5.3-fold increase in potency in total T cells, a 5.6-fold increase in B cells, and a 4.3-fold increase in monocytes compared with the JAK1,2,3 inhibitors measured (**Figure 4A**). Upadacitinib was found to have similar potency to deucravacitinib in the pSTAT2 assay compared with less potent tofacitinib and baricitinib. A striking difference in potency was observed in a functional assay of IFNα-activated monocytes (**Figure 4B**). Deucravacitinib was observed to be 7-fold, 13-fold, and 60-fold more potent than upadacitinib, baricitinib, and tofacitinib, respectively, showing a difference between targeting TYK2 over JAK1 in the Type I IFN pathway (**Table 3**). IFNγ is a Type II IFN with both overlapping and distinct yet critical functions from Type I IFN (44). Additionally, the IFNγ receptor pairs with JAK1/JAK2 to initiate downstream signaling and therefore is not an immediate target of deucravacitinib. Activation markers of monocytes and B cells induced by IFNγ, rather than IFNα, were not inhibited by deucravacitinib at relevant concentrations, indicating that it is a functional inhibitor of Type I IFN but not of Type II IFN (**Figure 4B**; **Supplementary Figure 2**). CXCL10 production in IFNα-treated monocyte-derived dendritic cells similarly shows a high preferential bias toward inhibition of TYK2 over JAK1. Interestingly, CXCL9, a related IFN-regulated chemokine, shows less of a TYK2-to-JAK1 bias in these same conditions (**Figure 4C**). Additional measures of chemokine production in total PBMC (**Table 3**) support observations of functional differences in TYK2 and JAK targeting within Type I IFN signaling. IL-12 and IL-23 are proinflammatory cytokines important for driving the maintenance of dysfunctional T-helper type 1 (Th1) and Th17 immune responses in certain inflammatory diseases (45–48). IL-12 and IL-23 signal through TYK2 via their shared p40 subunit, and JAK2 signals via p35 or p19, respectively. These cytokines are targets of deucravacitinib and baricitinib but not of tofacitinib or upadacitinib. IL-12 is a key regulator of IFNγ production. Previous works report deucravacitinib to show greater potency than JAK1,2,3 inhibitors against IL-12 + IL-18 (24). In this study, we find deucravacitinib to be highly potent and differentiated from JAK1,2,3 inhibitors against IL-12–, IL-12 + IL-18–, and IL-23–induced IFNγ production. In contrast, JAK1,2,3 inhibitors showed a range of potencies, from high to no potency against IL-12 and IL-23, possibly due to both on-target JAK1 and off-target JAK2 effects (**Figures 4D, E**; **Table 3**). IL-12–induced effects are largely thought to be synonymous with its downstream effector cytokine IFNγ whose effect is potentiated by JAK1/JAK2. Indeed, certain effects of IL-12 appear to be entirely driven by IFNγ, including chemokines that directly correlate with quantities of IFNγ produced (**Supplementary Figure 3**). However, we observed other inflammatory effects specifically driven by IL-12 signaling and independent of IFNγ (**Figure 4F**). **Figure 4G** shows IL-12–specific potency of deucravacitinib relative to JAK1,2,3 inhibitors, including a more than eight-fold increase in potency advantage of targeting TYK2 (deucravacitinib) than JAK2 (baricitinib) (**Table 3**).

**Table 3:** Potencies of inhibitors on inflammatory cytokines.

Kinase Pairing	Cytokine Stimulation	Endpoint	Compound	IC_50_ (nM)	95% CI (nM)	*P* value	Calculated WB IC_50_ (nM)	n
JAK1/TYK2	IFNα	mDC CXCL9 production	Deucravacitinib	3	1-10	NA	21	5
			Tofacitinib	52	38-72	0.0022	102	5
			Baricitinib	23	10-50	0.0131	51	5
			Upadacitinib	6	3-12	0.2094	12	5
		mDC CXCL10 production	Deucravacitinib	8	4-17	NA	55	5
			Tofacitinib	323	158-662	0.0008	635	5
			Baricitinib	179	79-408	0.0023	401	5
			Upadacitinib	52	25-108	0.0004	108	5
		Monocyte CD86 expression	Deucravacitinib	16	10-28	NA	110	6
			Tofacitinib	983	618-1564	0.0001	1934	6
			Baricitinib	219	113-421	0.0012	490	6
			Upadacitinib	116	52-257	0.0002	240	6
		PBMC CXCL10 production	Deucravacitinib	8	6-12	NA	55	6
			Tofacitinib	95	45-201	0.0001	187	6
			Baricitinib	29	10-88	0.0169	65	6
			Upadacitinib	17	7-38	0.0411	35	6
		PBMC CXCL11 production	Deucravacitinib	3	1-7	NA	21	6
			Tofacitinib	43	18-99	0.0001	85	6
			Baricitinib	15	9-26	0.0002	34	6
			Upadacitinib	6	2-17	0.1853	12	6
JAK2/TYK2	IL-12	PBMC IFNγ production	Deucravacitinib	21	13-35	NA	144	6
			Tofacitinib	259	44-1543	0.0063	510	5
			Baricitinib	165	71-387	0.0007	369	6
			Upadacitinib	68	15-300	0.155	141	5
	IL-12 + IL-18	NK-cell CD25 expression	Deucravacitinib	9	3-33	NA	62	6
			Tofacitinib	369	32-4244	0.0392	726	4
			Baricitinib	172	73-407	0.0284	385	5
			Upadacitinib	38	7-213	0.1119	79	6
		NK-cell IL18Rα expression	Deucravacitinib	30	19-48	NA	205	5
			Tofacitinib	546	66-4499	0.0507	1074	6
			Baricitinib	257	60-1103	0.0171	575	6
			Upadacitinib	193	58-641	0.0161	400	6
		PBMC IFNγ production	Deucravacitinib	16	22372	NA	110	6
			Tofacitinib	2237	889-5629	0.0007	4401	6
			Baricitinib	388	221-681	0.0034	868	6
			Upadacitinib	275	93-809	0.0123	570	6
	IL-1β + IL-23	PBMC IFNγ production	Deucravacitinib	9	6-15	NA	62	6
			Tofacitinib	138	12-1572	0.0188	272	6
			Baricitinib	96	27-342	0.0018	215	6
			Upadacitinib	120	53-271	0.0002	249	6
JAK1/JAK2	IFNγ	Monocyte CD86 expression	Deucravacitinib	526	356-776	NA	3314	6
			Tofacitinib	169	133-215	0.0018	338	6
			Baricitinib	67	56-79	0.0001	154	6
			Upadacitinib	49	35-68	0.0001	110	6

CI, confidence interval; IFN, interferon; IL, interleukin; JAK, Janus kinase; mDC, myeloid dendritic cell; NA, not applicable; NK, natural killer; PBMC, peripheral blood mononuclear cell; TYK2, tyrosine kinase 2, WB, whole blood.

## 4 Discussion

The introduction of JAK1,2,3 inhibitors has advanced autoimmune disease treatment but raised safety concerns due to lack of selectivity and resulting off-target effects among approved inhibitors. EPO, TPO and GM-CSF, essential for hematopoiesis, thrombopoiesis and myelopoiesis signal through JAK2 (49–51). JAK2 inhibition, an effective strategy for treating malignancy, results in anemia and thrombocytopenia (49, 50). Clinical trials of JAK1,2,3 inhibitors (baricitinib, tofacitinib, upadacitinib) aimed at autoimmune indications show similar effects, including platelet modulation and reductions in erythrocytes and neutrophils (9, 11, 52–54).

Deucravacitinib targets TYK2 through an allosteric mechanism by binding to the regulatory domain, which avoids the catalytic domain’s active site shared with other JAKs (55). Clinical trials of deucravacitinib have not shown clinical laboratory changes or safety signals indicative of JAK2 inhibition (26, 27, 56, 57), nor do *in vitro* STAT signaling assays reveal any potency against TPO or other JAK2-dependent cytokines (24, 25). JAK2 signaling assays for tofacitinib, baricitinib, and upadacitinib have produced variable potency results (24, 28, 29, 58–60), complicating the pharmacokinetic prediction of daily JAK2 inhibition (28, 30, 58).

Reductions of erythrocytes, platelets and granulocytes observed in clinical trials of JAK1,2,3 are likely due to decreased production. *In vitro* evaluations of JAK2/STAT signaling inhibition have focused on cell lines or non-proliferative mature myeloid cells and granulocytes and may not be accurate models of blood cell count abnormalities in patients. Instead, as a better model of clinical JAK2 inhibition liability, we have elected to use human pluripotent stem cells (HPSCs) which differentiate into specific lineages in response to EPO, TPO, G-CSF, stem cell factor, and GM-CSF (61–64). Deucravacitinib had no effect on megakaryocyte differentiation, consistent with stable clinical platelet count. JAK1,2,3 inhibitors affect megakaryocyte differentiation and their rank order (baricitinib > upadacitinib > tofacitinib) was consistent with the potency against JAK2 and inhibition in STAT assays.

Clinical studies show that tofacitinib decreases platelets that stabilize over time (52), while baricitinib and upadacitinib show an increase during the first month of treatment followed by a sharp decrease and eventual stabilization (13, 53, 65, 66). TPO levels in blood are balanced by platelet binding (67). Low platelet levels create excess TPO, which causes bone marrow HPSC to differentiate into megakaryocytes and produce more platelets (68). Hence, differences between tofacitinib, baricitinib and upadacitinib are likely due to complex TPO and megakaryocyte biology as well as differing drug distribution between bone marrow and blood.

Deucravacitinib does not affect neutrophils or red blood cell counts (26, 27, 56), while JAK1,2,3 inhibitors do impact these populations. In clinical trials baricitinib and upadacitinib reduce hemoglobin and neutrophils (12, 13), while tofacitinib reduces neutrophils but shows hemoglobin reduction only at higher doses (6, 69, 70). These differences are likely due to varying potency against bone marrow progenitor cells and the contribution of inhibiting JAK1 cytokines IL-6 and OSM to neutropenia and thrombocytopenia (71–73). Overall clinical, and *in vitro* data support that deucravacitinib lacks JAK2 activity, whereas tofacitinib, baricitinib, and upadacitinib exhibit JAK2 inhibitory activity *in vivo*.

Reductions in NK and T cells, increases in B cells, and lymphopenia are on-target effects of JAK1,2,3 inhibitors (74, 75) but not deucravacitinib (56). Hematopoietic dependency is attributed to specific cytokines, while lymphocyte homeostasis is likely influenced by all common gamma chain cytokines. The relative contribution of each of these toward lymphocyte populations have been determined through studying genetic deficiencies. IL-7 is crucial for thymic T cell development and peripheral maintenance (76), while a defective IL-7 receptor presents as T-cell–deficient (but not NK-cell–deficient) severe combined immunodeficiency (77). IL-2 supports various lymphocyte subtypes, but its loss results in lethal autoimmunity (78). IL-15 is vital for NK cell expansion and maturation (32), and its absence results in a dramatic loss of mature NK cells and memory CD8 T cells (79).

We chose functional assays of NK and CD8 proliferation and activation as a model of IL-15 effect. NK cells were considerably less prone to inhibition from kinase inhibitors than CD8 cells. Differences in kinase inhibitor potency for the same cytokine have been previously reported between cell types in pSTAT assays (29, 30) and our results indicate these differences extend to cell lineage subsets. Naive T cells were more resistant to kinase inhibition than effector and memory T cells in IL-7–induced BCL-2 assays. Both naive and memory T cells depend on IL-7 for survival, with naive cells undergoing apoptosis without IL-7 and memory cells experiencing partial reduction (1, 80). A lower threshold of JAK signaling required for naive versus memory cell survival may reflect how critical the pathway is to T-cell function. The differential effects on naive and memory populations in JAK1,2,3 inhibitor clinical trials remain unknown.

Signaling assays of IL-7 and IL-15 indicate that JAK1,2,3 inhibitors are highly potent, as previously reported with IL-2. Modeling based on IL-2 IC_50_ predicts up to 94% average daily inhibition at clinical dosage (28), which would suggest severe lymphopenia to be more prevalent with JAK1,2,3 inhibitor use. It is instead likely that functional measures of cytokine signaling, such as BCL-2 employed herein, are more predictive of *in vivo* biological pharmacodynamic effects. In psoriasis clinical trials, T-cell or NK-cell counts remained stable with deucravacitinib treatment (56), which is in agreement with a lack of effect on this pathway. Conversely, JAK1,2,3 inhibitor trials showed short-term fluctuations, followed by fast declines in NK and slower declines in CD4, and CD8 cells (74, 75). NK cells turn over three to four times faster than T cells (81), which may explain this observation and suggests different cytokine dependencies.

Tregs critically rely on IL-2 for survival and function (3, 41). This work shows deucravacitinib to minimally affect IL-2 and thus spare Tregs, unlike JAK1,2,3 inhibitors whose strong IL-2 potency could explain the decline in circulating peripheral Tregs observed in clinical trials of tofacitinib and baricitinib (75, 82). JAK1,2,3 inhibitors have also been associated with increased risk of serious infections, including herpes zoster (66, 70, 83). Infection rate in baricitinib trials was associated with decreased NK and CD4 T-cell counts (75), whereas for tofacitinib correlates were seen with overall lymphocyte count decreases but no particular subset (74). A similar infection profile is observed during Type I and Type II IFN inhibition when a very high pathway blockade is achieved (84, 85).

The overall infection risk of JAK1,2,3 inhibitors can likely be attributed to broad immunosuppression involving many cytokines and lymphocyte decreases. Deucravacitinib is a potent Type I IFN inhibitor and indirectly inhibits IFNγ; however, infection rates are low relative to JAK1,2,3 inhibitors due to optimized dosing that sufficiently controls inflammation without complete pathway inhibition (26, 28). This is consistent with TYK2 partial loss of function mutations known to be protective against autoimmunity without evidence of susceptibility to infection (86, 87).

Functional assays provide additional value over signaling assays alone. Inhibition of STAT signaling from JAK-regulated cytokines varies between cell types, and non-trivial differences within the same population have been reported (24, 30). Discerning the relative signaling contribution of different STATs is complex and not understood. Deucravacitinib and JAK1,2,3 inhibitors inhibit Type I IFN via TYK2 and JAK1, respectively, with deucravacitinib being more potent. How much more potent depends on the cell type and STAT assay (24). The functional data presented in this report reinforces this observation, while possibly being more meaningful in the context of observed pharmacodynamic effects *in vivo*. Herein, deucravacitinib was at least six-fold more potent against IFNα-induced CXCL10 than JAK inhibitors. Phase 2 SLE data shows deucravacitinib reduces CXCL10 by 42% at week 12 (88) and the IFN-5 (*MX1*, *HERC5*, *IFIT1*, *RSAD2*, *EIF2AK2*) gene signature score by up to 70% at Week 4 (89). Baricitinib reduced CXCL10 by 23% at week 12 and IFN-5 genes up to 32% in SLE patients (90, 91). For upadacitinib, CXCL10 levels fluctuated in SLE patients and did not reduce at week 12, whereas the IFN signature reduced by 29% but only at week 24 and not prior (92). Final evaluation of clinical efficacy for upadacitinib in SLE trials is ongoing. While final conclusions cannot be made from cross-trial comparisons, the data suggest that JAK1,2,3 inhibitors have a less robust effect against Type I IFN than deucravacitinib. It appears that IFN signaling has greater reliance on TYK2 than JAK1, which is in line with models showing the average coverage of JAK1 inhibition by baricitinib and upadacitinib to be as high or higher than deucravacitinib for TYK2 (28, 30).

Potency differences in shared pathways of TYK2 and JAK expand beyond Type I IFN. Baricitinib is equipotent against both JAK1 and JAK2 (31) and assays against common gamma chain cytokines conducted herein confirm high JAK1 potency. However, our assessment of IL-12 or IL-23 function show deucravacitinib is more potent than baricitinib by 20-fold in the TYK2/JAK2 pathway. This suggests that targeting TYK2 in IL-12/23–driven diseases such as psoriasis is more effective, while additionally avoiding JAK1,2,3 inhibitor hematopoietic safety concerns.

The mechanistic reasons for favoring TYK2 inhibition and the variability in functional readouts in specific cytokine pathways are unclear. JAK family member mutations can reduce or enhance cytokine signaling (93, 94), but effects vary by cytokine and cell type (86, 95, 96), suggesting fundamental differences in signaling requirements among JAK family members within receptor pairs. Other differences could be due to the targeting modalities. The pseudokinase domain lacks catalytic activity and regulates kinase signaling by steric inhibition of the kinase domain or by limiting catalytic site flexibility (97). Deucravacitinib inhibits TYK2 by stabilizing the structure of the pseudokinase domain in the inactive confirmation, thereby preventing the active site from having the mobility to phosphorylate (25). This is fundamentally different from tofacitinib, baricitinib, and upadacitinib, which are adenosine triphosphate (ATP) competitive active site inhibitors and work by blocking phosphorylation directly.

Potential limitations of this work include the use of healthy donor immune cells versus those sourced from psoriasis or SLE patients; however, as we investigated fundamental cellular processes, we believe these data to be highly valuable. Further work evaluating immune cell functionality, such as assessing Treg suppression ability, could be performed to expand on the chosen readouts of Treg markers, BCL2, and GARP expression. Killing assays could be performed to more deeply understand functional consequences of JAK1,2,3 inhibition on CD8 and NK cells. Additionally, our observation of pathway inhibition favoring TYK2 over JAK1/2 was limited to studying key inflammatory cytokines (Type I IFN, IL-12, and IL-23). This may or may not be observed in other common pathways (i.e., IL-19, IL-20, IL-22).

The data presented herein have shown the importance of functional analysis alongside signaling assays when studying kinase inhibitors. This work reinforces that deucravacitinib is TYK2 selective with superior target fidelity compared to approved JAK1,2,3 inhibitors, sparing JAK2 and JAK3 homeostatic pathways. Deucravacitinib is more potent in Type I IFN and IL-12/IL-23 pathways than JAK2 inhibitors. These findings support clinical observations that deucravacitinib effectively limits cytokine-driven inflammation in autoimmune disease, without compromising normal immune function.

## Data Availability

Bristol Myers Squibb details their data sharing request process via https://www.bms.com/researchers-and-partners/independent-research/data-sharing-request-process.html.
