# Supplementary material for: Nonclinical evaluations of deucravacitinib and Janus kinase inhibitors in homeostatic and inflammatory pathways
Source: Front Immunol. 2024 Sep 30;15:1437512. doi: 10.3389/fimmu.2024.1437512 (PMC11472182; doi:10.3389/fimmu.2024.1437512)

SUPPLEMENTARY MATERIAL

**SUPPLEMENTARY TABLE 1.** Potencies of inhibitors in JAK2 hematopoietic assays, inhibitors common to gamma chain cytokine assays, and inhibitors on inflammatory cytokines.

| **Kinase pairing** | **Cytokine stimulation** | **Endpoint** | **Compound** | **IC_50_ (nM)** | **95% CI (nM)** | ***P* value** | **Calculated WB IC_50_ (nM)** | **n** |
| --- | --- | --- | --- | --- | --- | --- | --- | --- |
| JAK2/JAK2 | IL-3, EPO,  G-CSF,  GM-CSF,  stem cell factor | BFU-erythroid | Deucravacitinib | >10000 | NA | NA | >10000 | 4 |
|  |  |  | Tofacitinib | 692 | 544-882 | 0.0001 | 1361 | 4 |
|  |  |  | Baricitinib | 150 | 118-189 | 0.0001 | 336 | 4 |
|  |  |  | Upadacitinib | 341 | 209-554 | 0.0002 | 706 | 4 |
|  |  | Total myeloid | Deucravacitinib | 4658 | 3115-6965 | NA | >10000 | 4 |
|  |  |  | Tofacitinib | 619 | 364-1054 | 0.0001 | 1218 | 4 |
|  |  |  | Baricitinib | 180 | 128-253 | 0.0004 | 403 | 4 |
|  |  |  | Upadacitinib | 208 | 94-460 | 0.0002 | 431 | 4 |
| JAK2/JAK2, JAK1/JAK2 | TPO, IL-3,  IL-6 | Medium CFU-Mk | Deucravacitinib | 2335 | 438->10000 | NA | >10000 | 4 |
|  |  |  | Tofacitinib | 1954 | 270- >10000 | 0.7257 | 3844 | 4 |
|  |  |  | Baricitinib | 538 | 287-1011 | 0.0219 | 1204 | 4 |
|  |  |  | Upadacitinib | 377 | 84-1697 | 0.013 | 781 | 4 |
| JAK1/JAK3 | IL-15 | CD8 T-cell pSTAT5 | Deucravacitinib | 465 | 251-862 | NA | 3184 | 6 |
|  |  |  | Tofacitinib | 14 | 3-63 | 0.0025 | 28 | 6 |
|  |  |  | Baricitinib | 6 | 1-34 | 0.0013 | 13 | 6 |
|  |  |  | Upadacitinib | 5 | 2-11 | 0.0001 | 10 | 6 |
|  |  | CD8 T-cell NKG2D expression | Deucravacitinib | 740 | 576-951 | NA | 5067 | 6 |
|  |  |  | Tofacitinib | 22 | 13-37 | 0.0001 | 43 | 6 |
|  |  |  | Baricitinib | 18 | 9-35 | 0.0001 | 40 | 6 |
|  |  |  | Upadacitinib | 14 | 7-29 | 0.0001 | 29 | 6 |
|  | IL-7 | Treg pSTAT5 | Deucravacitinib | 318 | 204-496 | NA | 2178 | 6 |
|  |  |  | Tofacitinib | 18 | 11-32 | 0.0001 | 35 | 6 |
|  |  |  | Baricitinib | 7 | 1-50 | 0.0032 | 16 | 6 |
|  |  |  | Upadacitinib | 7 | 3-17 | 0.0001 | 15 | 6 |
|  |  | Total CD4 T-cell proliferation | Deucravacitinib | 149 | 84-264 | NA | 1020 | 6 |
|  |  |  | Tofacitinib | 9 | 6-15 | 0.0001 | 18 | 6 |
|  |  |  | Baricitinib | 14 | 8-24 | 0.0004 | 31 | 6 |
|  |  |  | Upadacitinib | 7 | 4-14 | 0.0001 | 15 | 6 |
|  |  | Treg BcL-2 expression | Deucravacitinib | 592 | 451-777 | NA | 4054 | 6 |
|  |  |  | Tofacitinib | 56 | 47-67 | 0.0005 | 110 | 4 |
|  |  |  | Baricitinib | 38 | 22-66 | 0.0001 | 85 | 6 |
|  |  |  | Upadacitinib | 14 | 4-51 | 0.0023 | 29 | 4 |
|  |  | Naive CD4 T-cell proliferation | Deucravacitinib | 154 | 67-353 | NA | 1055 | 5 |
|  |  |  | Tofacitinib | 16 | 9-27 | 0.0027 | 32 | 6 |
|  |  |  | Baricitinib | 20 | 5-84 | 0.1976 | 45 | 4 |
|  |  |  | Upadacitinib | 16 | 4-64 | 0.0131 | 33 | 4 |

|  | IL-2 | Memory Treg expression | Deucravacitinib | 1738 | 706-4275 | NA | >10000 | 6 |
| --- | --- | --- | --- | --- | --- | --- | --- | --- |
|  |  |  | Tofacitinib | 19 | 8-46 | 0.0001 | 38 | 6 |
|  |  |  | Baricitinib | 8 | 4-17 | 0.0001 | 18 | 6 |
|  |  |  | Upadacitinib | 3 | 1-8 | 0.0001 | 6 | 6 |
|  |  | Naive Treg expression | Deucravacitinib | 887 | 441-1783 | NA | 6072 | 6 |
|  |  |  | Tofacitinib | 15 | 8-29 | 0.0001 | 29 | 6 |
|  |  |  | Baricitinib | 7 | 4-14 | 0.0001 | 16 | 6 |
|  |  |  | Upadacitinib | 2 | 2-3 | 0.0001 | 5 | 6 |
|  |  | Memory Treg  BCL-2 expression | Deucravacitinib | 2688 | 1325-5450 | NA | >10000 | 6 |
|  |  |  | Tofacitinib | 44 | 13-144 | 0.0003 | 86 | 6 |
|  |  |  | Baricitinib | 24 | 14-39 | 0.0001 | 53 | 6 |
|  |  |  | Upadacitinib | 11 | 9-13 | 0.0001 | 22 | 6 |
|  |  | Memory Treg GARP expression | Deucravacitinib | 781 | 487-1254 | NA | 5350 | 6 |
|  |  |  | Tofacitinib | 13 | 7-23 | 0.0001 | 26 | 6 |
|  |  |  | Baricitinib | 9 | 5-15 | 0.0001 | 19 | 5 |
|  |  |  | Upadacitinib | 5 | 2-11 | 0.0001 | 10 | 6 |
| JAK1/TYK2 | IFNα | Monocyte CD80 expression | Deucravacitinib | 10 | 5-19 | NA | 69 | 5 |
|  |  |  | Tofacitinib | 121 | 56-258 | 0.0018 | 238 | 6 |
|  |  |  | Baricitinib | 35 | 21-59 | 0.0076 | 78 | 6 |
|  |  |  | Upadacitinib | 21 | 15-29 | 0.0763 | 44 | 6 |
|  |  | Monocyte CD69 expression | Deucravacitinib | 7 | 5-9 | NA | 48 | 6 |
|  |  |  | Tofacitinib | 168 | 103-275 | 0.0001 | 331 | 6 |
|  |  |  | Baricitinib | 35 | 24-50 | 0.0002 | 78 | 6 |
|  |  |  | Upadacitinib | 38 | 20-71 | 0.0004 | 79 | 6 |
|  |  | B-cell CD40 expression | Deucravacitinib | 24 | 15-38 | NA | 164 | 6 |
|  |  |  | Tofacitinib | 134 | 34-519 | 0.024 | 264 | 6 |
|  |  |  | Baricitinib | 107 | 73-158 | 0.0005 | 239 | 6 |
|  |  |  | Upadacitinib | 56 | 18-171 | 0.0385 | 116 | 6 |
| JAK2/TYK2 | IL-12 | NK-cell CD25 expression | Deucravacitinib | 10 | 5-23 | NA | 69 | 6 |
|  |  |  | Tofacitinib | 287 | 2->10000 | 0.1334 | 565 | 5 |
|  |  |  | Baricitinib | 250 | 45-1386 | 0.007 | 559 | 5 |
|  |  |  | Upadacitinib | 117 | 11-1195 | 0.0142 | 242 | 5 |
|  |  | PBMC CXCL9 production | Deucravacitinib | 32 | 11-90 | NA | 219 | 5 |
|  |  |  | Tofacitinib | 32 | 15-68 | 0.9789 | 63 | 5 |
|  |  |  | Baricitinib | 20 | 10-43 | 0.4579 | 45 | 5 |
|  |  |  | Upadacitinib | 4 | 0.4-34 | 0.0857 | 8 | 5 |
|  | IL-12 + IL-18 | PBMC CXCL10 production | Deucravacitinib | 63 | 27-147 | NA | 431 | 6 |
|  |  |  | Tofacitinib | 208 | 152-285 | 0.01 | 409 | 6 |
|  |  |  | Baricitinib | 54 | 41-72 | 0.6841 | 121 | 6 |
|  |  |  | Upadacitinib | 35 | 24-52 | 0.1263 | 73 | 6 |
|  |  | PBMC CXCL11 production | Deucravacitinib | 30 | 11-83 | NA | 205 | 6 |
|  |  |  | Tofacitinib | 76 | 38-153 | 0.005 | 150 | 6 |
|  |  |  | Baricitinib | 17 | 10-29 | 0.1031 | 38 | 6 |
|  |  |  | Upadacitinib | 11 | 7-19 | 0.0251 | 23 | 6 |
|  |  | B-cell T-bet expression | Deucravacitinib | 302 | 195-469 | NA | 2068 | 6 |
|  |  |  | Tofacitinib | 165 | 120-227 | 0.0166 | 325 | 6 |
|  |  |  | Baricitinib | 36 | 24-54 | 0.0001 | 81 | 6 |
|  |  |  | Upadacitinib | 28 | 20-40 | 0.0001 | 58 | 6 |

| JAK1/JAK2 | IFNγ | Monocyte CD69 expression | Deucravacitinib | 342 | 203-575 | NA | 2342 | 6 |
| --- | --- | --- | --- | --- | --- | --- | --- | --- |
|  |  |  | Tofacitinib | 107 | 80-143 | 0.0006 | 211 | 6 |
|  |  |  | Baricitinib | 31 | 20-48 | 0.0002 | 69 | 6 |
|  |  |  | Upadacitinib | 18 | 10-34 | 0.0002 | 37 | 6 |
|  |  | B-cell CD40 expression | Deucravacitinib | 591 | 226-1542 | NA | 4047 | 6 |
|  |  |  | Tofacitinib | 306 | 181-520 | 0.0473 | 602 | 6 |
|  |  |  | Baricitinib | 50 | 23-111 | 0.0042 | 112 | 6 |
|  |  |  | Upadacitinib | 38 | 18-79 | 0.0042 | 79 | 6 |
|  |  | PBMC CXCL10 production | Deucravacitinib | 599 | 418-858 | NA | 4102 | 6 |
|  |  |  | Tofacitinib | 239 | 172-333 | 0.0054 | 470 | 6 |
|  |  |  | Baricitinib | 82 | 65-104 | 0.0001 | 184 | 6 |
|  |  |  | Upadacitinib | 44 | 28-67 | 0.0001 | 91 | 6 |
|  |  | PBMC CXCL11 production | Deucravacitinib | 223 | 116-430 | NA | 1527 | 6 |
|  |  |  | Tofacitinib | 94 | 57-157 | 0.0006 | 185 | 6 |
|  |  |  | Baricitinib | 33 | 19-55 | 0.0001 | 74 | 6 |
|  |  |  | Upadacitinib | 13 | 5-39 | 0.0001 | 27 | 6 |
|  |  | B-cell T-bet expression | Deucravacitinib | 719 | 567-912 | NA | 4924 | 6 |
|  |  |  | Tofacitinib | 115 | 68-194 | 0.0004 | 226 | 6 |
|  |  |  | Baricitinib | 44 | 31-62 | 0.0001 | 98 | 6 |
|  |  |  | Upadacitinib | 22 | 11-44 | 0.0001 | 46 | 6 |

BFU, burst forming unit; CFU-Mk, colony forming unit megakaryocyte; CI, confidence interval; EPO, erythropoietin; G-CSF, granulocyte colony stimulating factor; GM-CSF, granulocyte-macrophage colony-stimulating factor; IFN, interferon; IL, interleukin; JAK, Janus kinase; NA, not applicable; NK, natural killer; PBMC, peripheral blood mononuclear cell; pSTAT, phosphorylation of signal transduction and activation of transcription; TPO, thrombopoietin; Treg, regulatory T cell; TYK2, tyrosine kinase 2.

**SUPPLEMENTARY TABLE 2.** Potencies of kinase inhibitors on IL-7 signaling.

| **Assay** | **Cytokine stimulation** | **Endpoint** | **Compound** | **Measured IC_50_ (nM)** | **95% CI  (nM)** | **Calculated WB  IC_50_ (nM)** | **n** |
| --- | --- | --- | --- | --- | --- | --- | --- |
| PBMC | IL-7 | Total CD3 T-cell pSTAT5 | Deucravacitinib | 277 | 126-611 | 1897 | 6 |
|  |  |  | Tofacitinib | 19 | 8-47 | 37 | 6 |
|  |  |  | Baricitinib | 9 | 4-24 | 20 | 6 |
|  |  |  | Upadacitinib | 10 | 4-29 | 21 | 6 |
| WB | IL-7 | Total CD3 T-cell pSTAT5 | Deucravacitinib | 1960 | 1792-2137 | - | 4 |
|  |  |  | Tofacitinib | 38 | 32-44 | - | 3 |
|  |  |  | Baricitinib | 44 | 37-52 | - | 4 |
|  |  |  | Upadacitinib | 41 | 35-47 | - | 4 |

CI, confidence interval; IL, interleukin; PBMC, peripheral blood mononuclear cell; pSTAT, phosphorylation of signal transduction and activation of transcription; WB, whole blood.

**SUPPLEMENTARY FIGURE 1.** Representative examples of cell proliferation, molecule expression, and flow gating strategies. **(A)** Proliferation gating and functional marker NKG2D^+^ expression of NK cells and CD8^+^ T cells. **(B)** Treg cell lineage gating, functional marker GARP^+^ expression, and functional marker BcL-2 expression.. **(C)** CD4^+^ T cell lineage gating and signaling marker pSTAT5 expression. **(D)** Expression of functional marker CD40 on CD19^+^ B cells and expression of functional markers CD69, CD80, and CD86 on CD19^+^ monocytes. **(E)** Expression of functional marker CD25 and IL18Rα on NK cells and T-bet on CD19^+^ B cells. Representative donor histograms show response to a given concentration of deucravacitinib and tofacitinib, and unstimulated and cytokine stimulated controls for comparison.


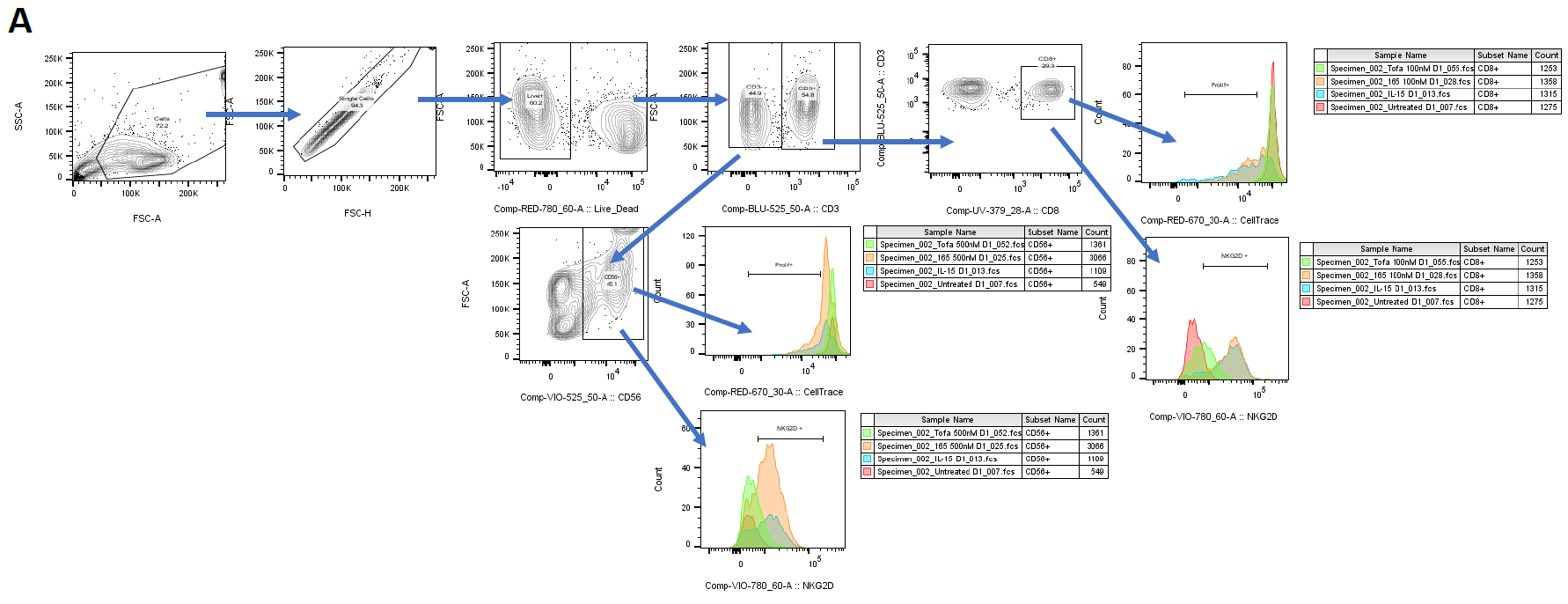


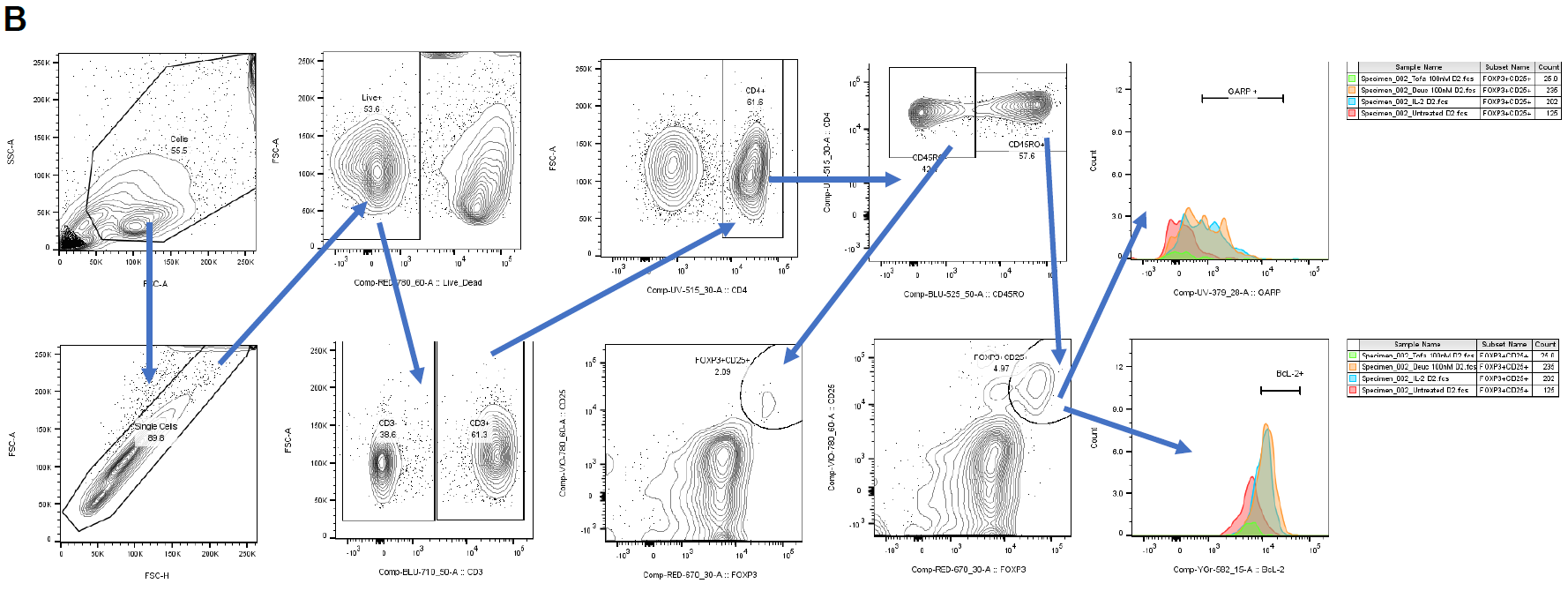


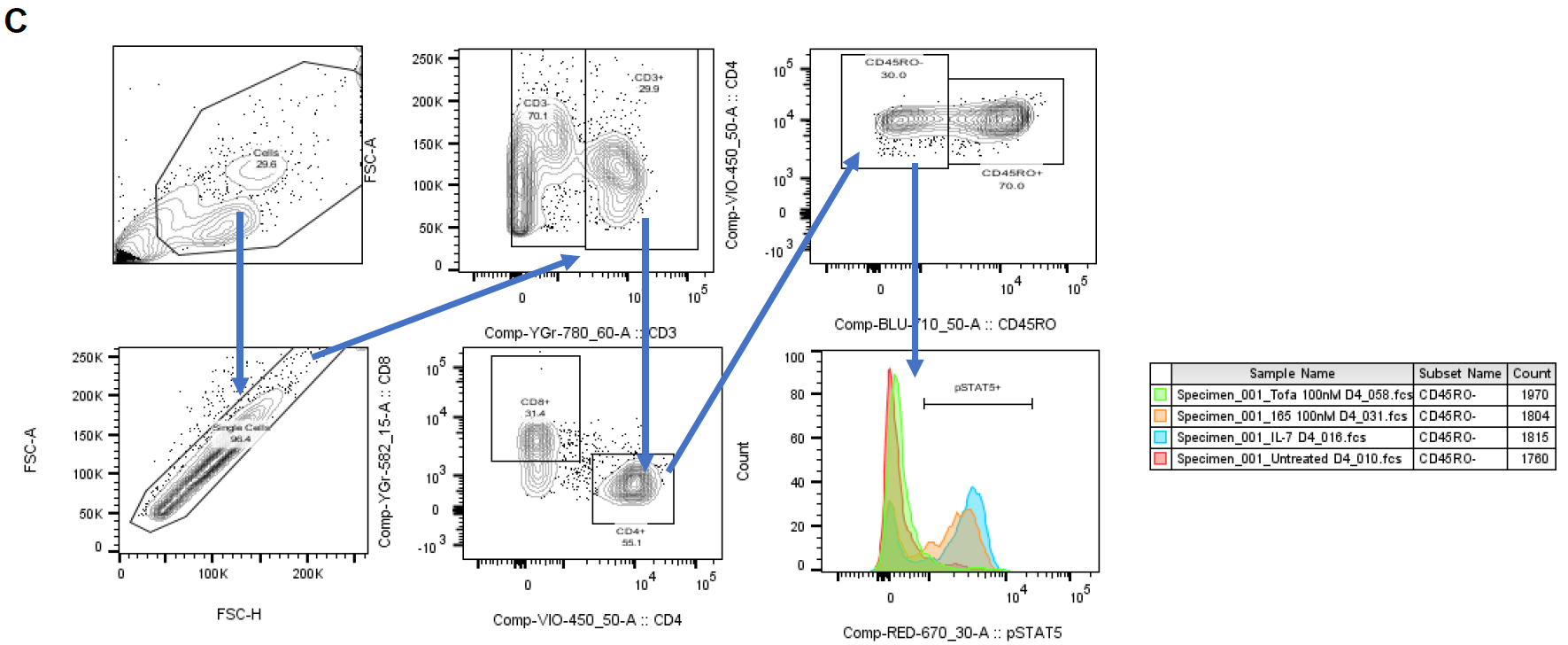


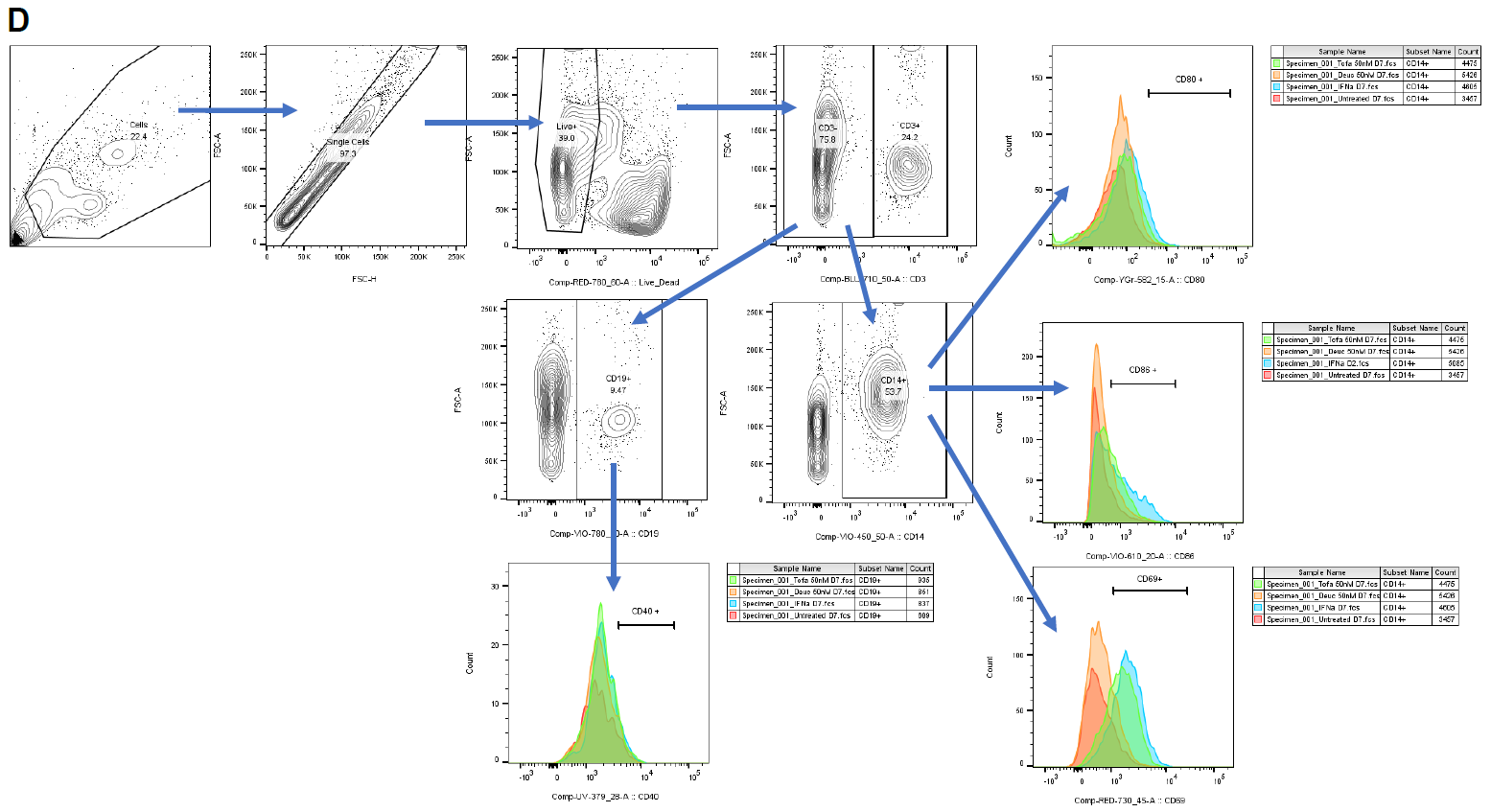


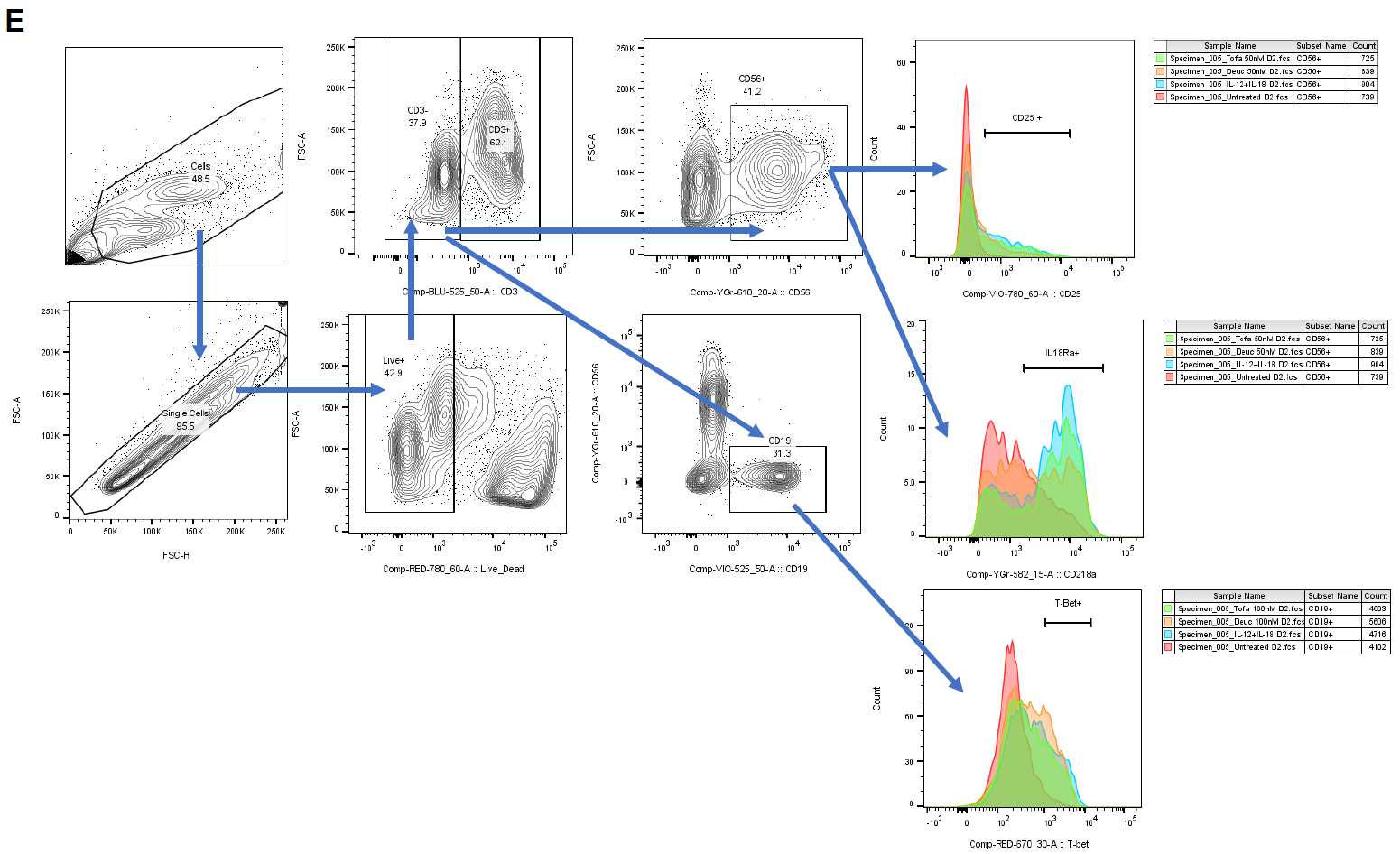


**SUPPLEMENTARY FIGURE 2.** Kinase inhibitors in functional assays of IFN. PBMCs from normal healthy volunteers were treated with IFNα (1000 U/mL) or IFNγ (25 ng/mL) overnight in the presence or absence of dose-ranging kinase inhibitor and measured for the inhibition of CD69 expression by median fluorescence intensity on CD14^+^ monocytes and CD40 expression by median fluorescence intensity on CD19^+^ B cells (n = 6 donors). Statistical significance from deucravacitinib was determined by paired two-tailed *t*-test. **P*<0.05, ***P*<0.01, ****P*<0.001.


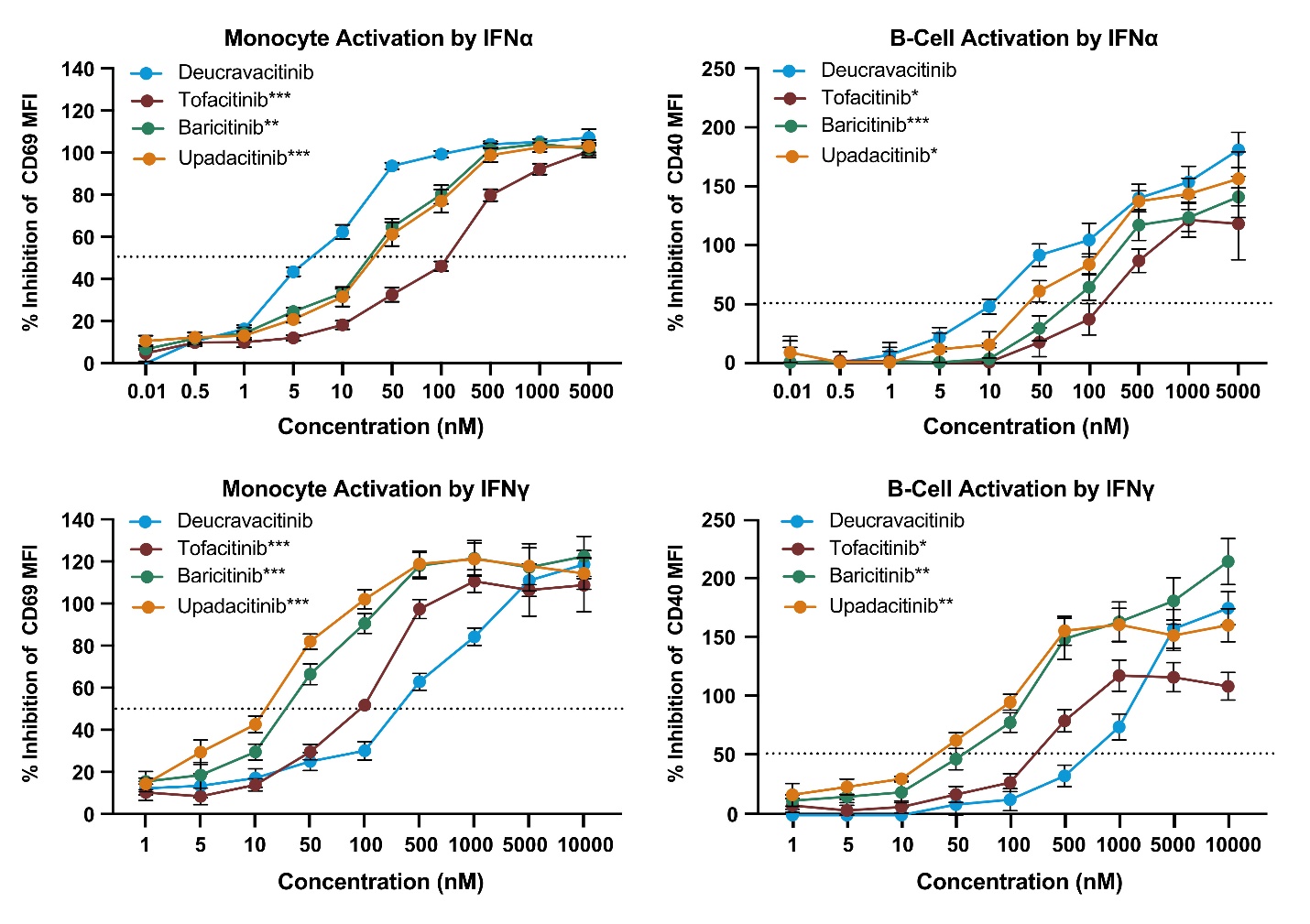


**SUPPLEMENTARY FIGURE3.** Kinase inhibitors against direct or indirect IFNγ functional response. (**A**) PBMCs from normal healthy volunteers were treated with IFNγ (25 ng/mL) or IL-12 (2 ng/mL) + IL-18 (5 ng/mL) overnight in the presence or absence of kinase inhibitors and measured for functional outputs and inhibitory response. (**B**) PBMCs from normal healthy volunteers were treated with IFNα (1000 U/mL) or IFNγ (25 ng/mL) overnight and measured for chemokine production. (C) PBMCs from normal healthy volunteers were treated with IL-12 (10 ng/mL) overnight. Donor chemokine production was correlated with measured IFNγ. Correlation was performed using a simple linear regression using Pearson correlation. Statistical significance for expression analysis was determined by one-way ANOVA and JAK1,2,3 from deucravacitinib was determined by paired two-tailed *t*-test. **P*<0.05, ***P*<0.01, ****P*<0.001, n = 5-6 donors.
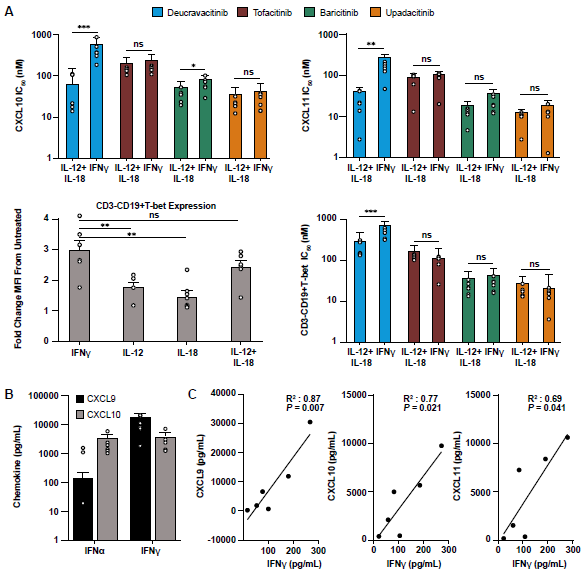

Supplement: Supplementary file 1 [file DataSheet1.docx]
